# Non-Coding RNAs in Brain Tumors, the Contribution of lncRNAs, circRNAs, and snoRNAs to Cancer Development—Their Diagnostic and Therapeutic Potential

**DOI:** 10.3390/ijms21197001

**Published:** 2020-09-23

**Authors:** Julia Latowska, Adriana Grabowska, Żaneta Zarębska, Konrad Kuczyński, Bogna Kuczyńska, Katarzyna Rolle

**Affiliations:** 1 Department of Molecular Neurooncology, Institute of Bioorganic Chemistry, Polish Academy of Sciences, 61-705 Poznań, Poland; jlatowska@ibch.poznan.pl (J.L.); agrabowska@ibch.poznan.pl (A.G.); zzarebska@ibch.poznan.pl (Ż.Z.); kuczynski@ibch.poznan.pl (K.K.); bkuczynska@ibch.poznan.pl (B.K.); 2 NanoBioMedical Centre, Adam Mickiewicz University, Wszechnicy Piastowskiej 3, 61-614 Poznań, Poland

**Keywords:** glioblastoma, glioma, medulloblastoma, brain tumors, lncRNA, circRNA, snoRNA, cancer hallmarks

## Abstract

Brain tumors are one of the most frightening ailments that afflict human beings worldwide. They are among the most lethal of all adult and pediatric solid tumors. The unique cell-intrinsic and microenvironmental properties of neural tissues are some of the most critical obstacles that researchers face in the diagnosis and treatment of brain tumors. Intensifying the search for potential new molecular markers in order to develop new effective treatments for patients might resolve this issue. Recently, the world of non-coding RNAs (ncRNAs) has become a field of intensive research since the discovery of their essential impact on carcinogenesis. Some of the most promising diagnostic and therapeutic regulatory RNAs are long non-coding RNAs (lncRNAs), circular RNAs (circRNAs), and small nucleolar RNAs (snoRNAs). Many recent reports indicate the important role of these molecules in brain tumor development, as well as their implications in metastasis. In the following review, we summarize the current state of knowledge about regulatory RNAs, namely lncRNA, circRNAs, and snoRNAs, and their impact on the development of brain tumors in children and adults with particular emphasis on malignant primary brain tumors—gliomas and medulloblastomas (MB). We also provide an overview of how these different ncRNAs may act as biomarkers in these tumors and we present their potential clinical implications.

## 1. Introduction

Every year, the global number of cancer cases reaches over 18 million, of which it is estimated that more than half result in death. Even though nervous system tumors only account for approximately 3% of all cancers in the world, they are considered to be one of the deadliest of all forms of cancer [1]. Despite intensive and continuous research, the mortality rate of neural system tumors is estimated to be 3.4 per 100,000 people in the world [2]. 

High-grade gliomas are characterized by uncontrolled proliferation, invasiveness, necrosis formation, lack of apoptosis, and dynamic angiogenesis [3]. These features, combined with difficult access to the tumor, mean that despite the combination of surgical treatment with radiotherapy and chemotherapy followed by Stupp protocol, only limited therapeutic success is expressed—the median survival in the case of patients diagnosed with glioblastoma (GBM)—the most aggressive type of brain cancer—is still less than two years [4]. Furthermore, with pediatric brain tumors, after entering adulthood, child survivors struggle with the long-term consequences of subjecting their developing brain to medical procedures such as surgery, radiotherapy, and/or chemotherapy [5]. One of the reasons for this failure is that the molecular mechanisms responsible for the growth and invasion of brain cancer are still poorly understood. Additionally, these cancers possess unique properties connected with the individual developmental, microenvironmental, genetic, and epigenetic features of the brain [6,7,8]. Thus, there is an urgent necessity to deepen the knowledge about the molecular mechanisms governing tumor progression and to develop more effective clinical strategies for cancer treatment.

During the development of next-generation sequencing methods, it becomes evident that the coding part of the genome constitutes a minor fraction of the whole genetic information. Large-scale genome-wide sequencing has revealed the tissue-specific expression of non-coding RNAs (ncRNAs) and their essential importance as crucial regulators in fundamental biological processes [9]. The diverse roles of ncRNAs include regulating gene transcription, post-transcription, translation, and epigenetic modification [10,11]. In addition, it is being increasingly reported that specific ncRNAs are linked with cancers, including brain tumors. They can affect many processes related to the development of cancer, including cell proliferation, apoptosis, migration, invasion, maintenance of stemness, and even tumor microenvironment remodeling and tumor metastasis [12,13,14]. The best known and examined group of ncRNA remains microRNAs. In brain tumors, the level of microRNAs (miRNAs) is frequently disrupted. Many miRNAs have been identified as factors involved in tumor formation and propagation by regulating both oncogenes and tumor suppressors, which makes them a promising therapeutic target [15,16]. Many excellent reviews already summarized the miRNA function in brain tumors [15,16]. Hence, in this article, we present the collected state of knowledge covering long non-coding RNAs (lncRNAs), circular RNAs (circRNAs), and small nucleolar RNAs (snoRNAs) in adults and adolescent brain primary tumors such as gliomas and medulloblastomas, considering their impact on, and from the perspective of, the most basic mechanism of cancer development. We also make an effort to summarize the present knowledge of the ability of ncRNAs to be prognostic, diagnostic, and therapeutic molecules in brain tumors. 

## 2. Characterization of Selected Brain Tumors 

### 2.1. Adult Gliomas

Brain tumors constitute the majority of all primary central nervous system (CNS) tumors, of which approximately 80% are gliomas originating from glia, which present a relevant role in nourishing nerve cells and in facilitating impulse transmission [17].

The standard diagnostic procedure in the case of CNS tumors is initially based on computer tomography (CT) or more sensitive magnetic resonance imaging (MRI). Depending on the tumor location, as a result of increased intracranial pressure, the most frequent symptoms of brain tumors are headaches and dizziness, ataxia, blurred vision, frequent syncope, and epileptic seizures. Because of these nonspecific symptoms, brain tumors are often misdiagnosed as infections, inflammatory processes, or immune diseases. Current treatment options for brain tumors in most locations include maximal surgical resection, within the constraints of preserving the neurologic function and underlying health of patients. Pursuing radiotherapy and chemotherapy with temozolomide (TMZ), which shows the highest cancer survival rate in patients, has a major role in the treatment of high-grade gliomas [18,19]. Despite advanced imaging techniques, diagnostic methods still involve histopathological assessment after a tumor biopsy or surgical extraction [20].

The etiology of gliomas has not been thoroughly revealed; however, the established risk factors include genetic background, head injuries, or exposure to vinyl chloride, pesticides, polycyclic aromatics, and solvents [21]. A number of factors affect the aggressiveness of a tumor and determine a patient’s survival chances, namely tumor size and location, age at diagnosis, and histologic and genetic markers [22]. 

### 2.2. Glioblastoma

Glioblastoma (GBM) is the most prevalent malignant brain tumor in adults, located within the cerebral hemispheres, brainstem, or cerebellum. Due to the rapid growth and brain parenchymal infiltration, combined with resistance to treatment, GBM belongs amongst the most lethal of all cancers [23]. The majority (approximately 90%) of cases of GBM originate de novo while the remaining 10% are secondary malignancies that develop through the progression of lower-grade gliomas (diffuse or anaplastic astrocytomas), which last approximately four to five years [24]. 

Despite radical treatment, the median survival of these cancer patients is still low—approximately 14.6 months among patients with newly diagnosed cancer, with only 5% having a survivability of five years [4]. Meanwhile, untreated GBM cases lead to the death of the patient within three months as a result of increased intracranial pressure [25]. Such a low survival rate is due to both the high level of tumor invasion of adjacent tissues, preventing its complete removal and causing GBM recurrence, as well as the extremely high heterogeneity observed both between patients and within the tumor [23].

Histopathologically, GBM is characterized by cellular pleomorphism, mitosis, invasiveness, angiogenesis, and necrosis [26]. Currently, there are a wide range of molecular biomarkers, which are commonly tested as part of the molecular diagnostic of GBM patients. Notably, within gliomas, we can divide these molecular biomarkers into those with a point mutation in the IDH1 or IDH2 gene (IDH-mutant), which occurs predominantly in lower-grade gliomas (WHO grade II/III), or else into secondary GBM with no mutation (IDH-wildtype). These are primary mutations and are probably initiated in gliomas. The presence of the IDH1 mutation is associated with comparatively prolonged patient survival; the median overall survival in these patients is 3.8 years, and in patients without IDH mutation, survival is 1.1 years [27,28]. In IDH-wildtype GBM, the TERT promoter mutation is also observed [24,27].

The development of GBM is associated with the deregulation of the transition checkpoint from the G1 phase to the S phase in the cell cycle. In GBM, mutations within the gene encoding the tumor suppressor protein TP53 and a protein belonging to the family of chromatin remodeling proteins, ATRX, are most often observed, probably affecting the regulation of gene expression [27]. Sequencing of the GBM genome has revealed that in 90% of cells classified as GBM, part of the structure or the whole structure of chromosome 10 is lost. In addition, approximately 60% of all gliomas have an amplified gene coding epidermal growth factor receptor (EGFR), which, by binding to epidermal growth factor (EGF), contributes to its increased activity and promotes proliferation and intracellular signaling disorders [29]. Additionally, one of the most significant tumor suppressors—PTEN, located at 10q23.3—is often mutated or absent due to chromosomal alterations. Moreover, in 40% of cases of GBM, a deletion is observed in the CDKN2A gene, which encodes the p16INK4a protein—an inhibitor in the cell cycle [30].

Epigenetic mechanisms are considered to have a possible prognostic significance in GBM, the most essential of which is incorrect methylation of the CpG islands observed within the suppressor genes, which, consequently, may lead to carcinogenesis [31]. Likewise, methylation of the O6-methylguanine-DNA methyltransferase (MGMT) promoter has prognostic significance in patients, which occurs in approximately 50% of gliomas, and definitely more often in GBM recurrence [24]. Methylation of the MGMT promoter results in its reduced expression, making chemotherapy with alkylating agents more effective in these patients [32].

Based on gene expression patterns, four molecular subclasses of GBMs have been identified, namely proneural, classical, mesenchymal, and neural [33]. Nevertheless, to fully understand the process of the onset and progression of GBM oncogenesis, as well as the difficulties associated with the validation of biomarkers in clinical use, drug resistance, and treatment failure, recent studies have focused on searching for the ncRNAs associated with the above-mentioned subtypes. It has been proved that there are many ncRNAs whose expression patterns allow to distinguish clinical and molecular types of brain tumors, and even to apply them for determining survival prognosis [34].

Furthermore, it is necessary to take into account the role of the microenvironment of GBM, which displays heterogeneity in the cellular composition that contains a small subpopulation of tumor cells harboring stem-like properties [35,36]. Moreover, it is widely known that cancer cells can communicate with each other and can regulate progression through exosomes secreting, which also may be observed in gliomas and can be used as potential biomarkers [37].

### 2.3. Pediatric Gliomas

In the case of adolescents, CNS tumors are one the most common types of cancers, with high mortality rates [38]. Pediatric glioblastoma (pGBM) is characterized by slightly different properties and molecular patterns compared to that in adults. Somatic histone 3 mutations are observed in more than 50% of pGBMs, namely H3.3 K27M, characteristic of diffuse midline high-grade gliomas, and H3.3 G34R/V, seen in hemispheric high-grade gliomas. Mutations in IDH1 and IDH2 are observed in less than 5% of pediatric high-grade gliomas. Further, histone 3 and IDH1/2 wild-type pGBM can be subdivided into three subgroups: (1) receptor tyrosine kinase, characterized by the deletion of *CDKN2A/B*, the mutation of *TP53*, and the amplification of *EGFR* and *PDGFRA*; (2) mesenchymal, characterized by mutations in *NF1*; and (3) pleomorphic xanthoastrocytoma (PXA)-like, characterized by the deletion of *CKDN2A* and *BRAF* V600E mutations. Additionally, in 80% of pediatric high-grade gliomas, activation of the PI3-kinase/Akt/mTOR signaling pathway is observed, and the majority have mutations in the tumor suppressor gene *TP53.* Overexpression of p53 in these gliomas is correlated with a poorer five-year progression-free survival [39,40].

Many pediatric low-grade gliomas are characterized by genetic alterations associated with the RAS–MAPK pathway, the majority of which contain the BRAF oncogene. Tandem duplication on chromosome 7q34 occurs in 70–80% of pediatric pilocytic astrocytomas. These duplications result in a fusion of BRAF with KIAA1549. Another BRAF-related mutation observed in supratentorial hemispheric pilocytic astrocytomas, gangliogliomas, PXA, and dysembryplastic neuroepithelial tumor (DNET) is BRAF V600E. The occurrence of this mutation is associated with a worse prognosis among patients compared to that in the case of the BRAF–KIAA1549 fusion. It was discovered that another MAPK pathway had mutations in low-grade gliomas, for instance, FGFR1 alterations and NTRK family fusions, and changes in MYB and MYBL1 oncogenes in angiocentric glioma and diffuse astrocytomas (WHO grade II) [41,42]. In diffuse astrocytomas and PXA, CDKN2A/B mutations are sometimes observed [41]. Hereditary germline mutations also have an impact on the development of pediatric low-grade gliomas. The variation in TSC or NF1—a tumor suppressor gene encoding neurofibromin (a protein involved in the RAS–MAPK signaling and mTOR pathways)—has also been determined [38]. 

### 2.4. Medulloblastoma

Medulloblastoma (MB) is the most common highly aggressive malignant brain tumor of the CNS in children. It is speculated that the tumor develops from neural stem cell precursors in the granular cell layer of the cerebellum. Despite the multimodal therapeutic regimens that involve maximal surgical resection followed by radiotherapy and chemotherapy, the mortality rate of this tumor is still high, which is undoubtedly due to high tumor heterogeneity [43,44].

Taking into consideration specific transcriptional profiles, genetics, demographics, recurrence patterns, and outcomes, four subgroups of this cancer have already been distinguished: WNT, Sonic Hedgehog (SHH), group C, and group D [45]. The WNT subgroup is characterized by excellent prognosis and develops primarily in non-infant children. These tumors are associated with the activation of the WNT pathway, and are characterized by frequent mutations in exon 3 of CTNNB1 encoding b-catenin and monosomy 6. SHH tumors occur across all ages with an intermediate prognosis. They harbor mutations in Patched 1, SUFU, and Smoothened, correlating with the activation of the SHH pathway. This subgroup is also characterized by amplifications of GLI2 and MYCN and mutations in the TERT promoter. Subgroup C medulloblastoma is characterized by poor prognosis and develops in infants and young children. It is also associated with amplifications of the MYC oncogene. However, the most frequent subgroup, which contains 40% of cases, is group D medulloblastoma, which is characterized by amplification of MYCN, tandem duplications of SNCAIP, and mutations in chromosome 17 in 80% of cases [46,47].

## 3. Brief Insight into the World of Non-Coding RNAs 

**Figure 1:**
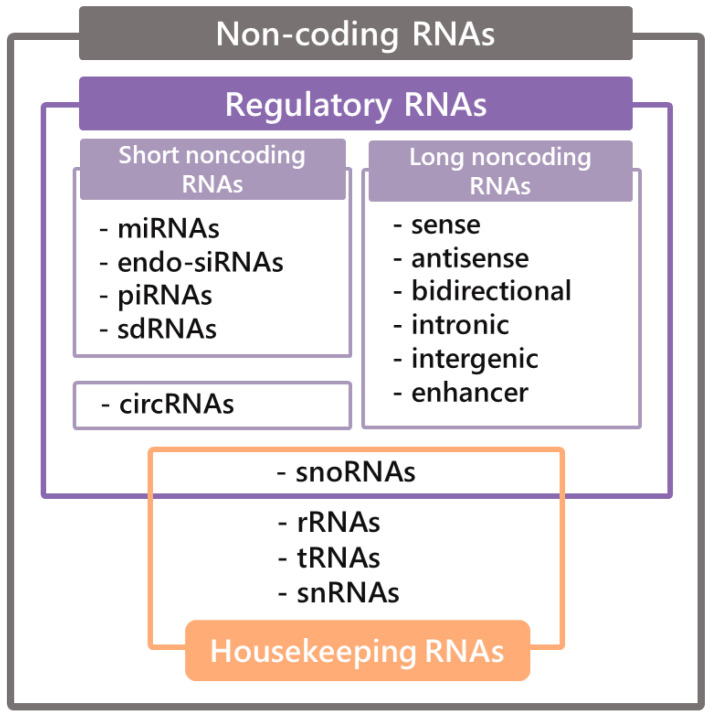
Classification of non-coding RNAs.

The history of ncRNAs dates back to 1965, when the first report elucidating the structure of alanine tRNA was provided [48]. This finding was followed by the subsequent discovery of snoRNAs, lncRNAs, miRNAs, and many more [49]. The application of the high-throughput sequencing method allowed the discovery of a wide range of ncRNAs and the conclusion that they constitute the majority of all genetic information. The classification of ncRNAs includes two groups, namely housekeeping RNAs, which are essential for maintenance of basic cellular function and constitutively expressed and regulatory RNAs. Regulatory RNAs can be further divided, depending on their length, into short ncRNAs for transcripts shorter than 200 nt and long ncRNAs, which are more than 200 nt in length (Figure 1) [50].

During the development of new molecular tools that made the exploration of the structures, functions, and cellular location of ncRNAs possible, it became clear that these particles play a significant role in cancer progression. The best known and examined group of ncRNAs remains microRNAs, which can act both as oncogenes and as tumor suppressors by inhibiting the expression of the crucial genes in the pathways that supervise cell processes, including cell cycle, apoptosis, and migration [51]. When miRNAs highly overexpressed in different types of cancer (oncomiRs) were discovered, it soon became evident that there are many more essential groups of ncRNAs responsible for cancer progression, three of which are lncRNAs, circRNAs, and snoRNAs, as presented below.

### 3.1. LncRNAs

Recently, increased attention has been focused on lncRNAs, which were initially considered as transcriptional noise; however, a growing body of evidence indicates that they play many regulatory roles, which rely on their spatial arrangement, facilitating various structures, as well as on their molecular interactions with other molecules. Most lncRNAs are transcribed by RNA polymerase II, and thus they share similarities with messenger RNAs (mRNAs), such as 5′ cap and 3′ poly(A) tail, despite their lack of coding potential [52].

Regarding the loci of the lncRNAs in the genome, they are divided into seven subgroups (Figure 1). Sense lncRNAs comprise one or more exons of coding genes in contrast to antisense lncRNAs, which demonstrate a partial or overall complementarity to functional transcripts. Bidirectional lncRNAs are composed of promoters, which are part of protein-coding genes, but are transcribed in a contrary direction; likewise, enhancer lncRNAs are made of enhancer regions. Intronic lncRNAs are produced from the intron of genes; furthermore, subsequent intergenic lncRNAs are placed between the protein-coding regions of two conterminous genes and are transcribed autonomously [53].

**Figure 2:**
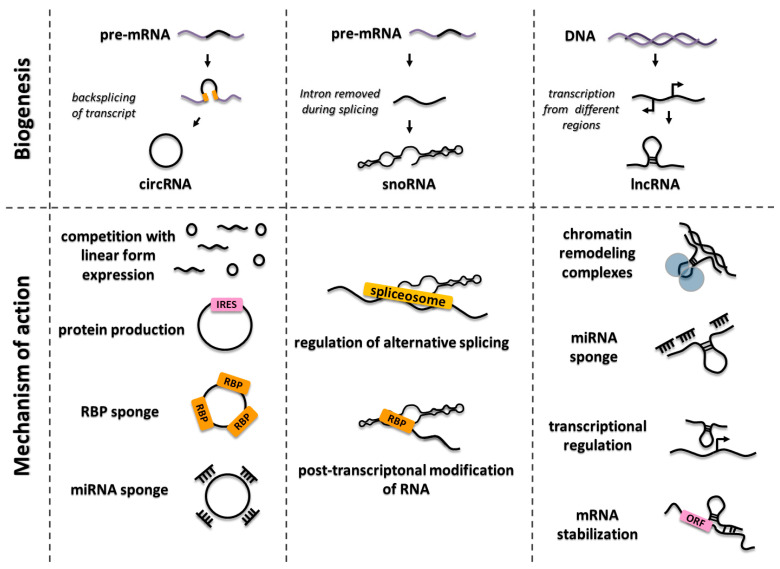
The principal functions of long non-coding RNAs (lncRNAs), circular RNAs (circRNAs), and small nucleolar RNAs (snoRNAs).

LncRNAs have been shown to perform diverse functions, including chromatin structure remodeling and histone modifications, to activate or repress genes in the nucleus or modulate signal transduction in cytosol [54,55]. Moreover, lncRNAs are involved in transcriptional regulation in a cis and trans manner, as well as in the post-transcriptional processing of other RNAs [56]. They can regulate cellular processes on various levels, for instance, by both activating and inhibiting gene expression. At the pre-transcriptional level, lncRNAs may function as epigenetic factors via cooperation with other chromatin-remodeling complexes (i.e., HOTAIR, ANRIL, and PARTICLE), and they can co-regulate transcriptional machinery as well (i.e., PANDA, RMST, and HOXA1). Some lncRNAs supervise gene expression level through post-transcriptional regulation, which can involve mRNA stabilization (HMMR-AS1), sequestering miRNAs, or recruiting proteins that repress translation (i.e., HCP5, KIAA0125, NRON, and lncRNA-p21) (Figure 2) [57,58]. 

Many lncRNAs play significant role in distinctive tumor development. For instance, a notable amount of data suggest that aberrant HOTAIR expression is associated with various types of cancer—for example, breast, hepatocellular, gastric, colorectal, and pancreatic cancers [59]. An additional distinguished oncogenic lncRNA associated with non-small cell lung cancer progression is MALAT1, which modulates cancer cell invasiveness [60]. Another lncRNA involved in oncogenesis is LED, which regulates the activity of P53 enhancers and indirectly leads to a decrease in the level of this protein [61].

### 3.2. CircRNAs

A distinctive feature that determines the uniqueness of circRNAs in comparison to the other forms of RNA molecules is the presence of a covalent bond joining the 3′ and 5′ ends, forming a continuous loop, preventing degradation by RNA exonucleases, followed by the lack of a 3′ poly(A) tail and a 5′ cap structure [62]. The presented features confer numerous properties, such as relatively high stability, which makes them exceptionally abundant in the cytoplasm. CircRNAs are generated from pre-messenger RNA via back-splicing [63]. Some of the circular transcripts might also appear via direct RNA ligation, circularization of introns that have escaped from debranching, or splicing of intermediates. Furthermore, two distinct types of circular transcripts have been proposed: exon-skipping events, characterized by internal splicing of skipped exons, which account for a small number of genes; and intron pairing-driven circularization, where intronic motifs such as Alu repeats promote closing of the molecule by positioning the splice sites in close proximity [64,65]. Many loci-containing circRNAs are surrounded by Alu-repeating elements. Such elements located within different introns of a given gene may pair with each other, leading to exon circularization. On the other hand, it has been proved that two Alu elements located within a single intron do not affect circRNA expression from a given gene. These data show that the distribution of the Alu sites that promote the circulation of a fragment that contains more elements than a single intron has a greater role in the circularization process than the number of Alu sites [66,67].

Cellular localization of the majority of circular transcripts might be directly linked with the function, mechanism of action, and following tissue specificity of the transcripts. However, the specific functions of several circRNAs and the modes of their action are still not understood well. Difficulties associated with the unequivocal determination of the function of circRNAs are related to the insufficient number of research protocols to precisely manipulate the expression level of circRNAs from their endogenous locus. Nowadays, computational methods serve as the most common approach in circRNA research, involving RNA-Seq with comprehensive algorithms as the most frequently used. The first characterized circRNA function, directly linked to the nucleotide sequence, is miRNA sponging. A well-known example of this type of interaction is described for ciRS-7/CDR1as—which is the antisense circular transcript of the CDR1 gene. A number of other circRNA functions have been elucidated, such as the potential impact on the regulation of alternative splicing by removing part of the pre-mRNA to produce circRNA in a back-splicing process, thus promoting the remaining fragment to splice or be degraded [67,68,69]. Moreover, circRNAs have been reported to interact with RNA binding proteins (RBPs), sequestering them and acting as a sponge. On the other hand, RBPs participate in the formation, translation, post-transcriptional regulation, and extracellular transport of circRNAs [70]. Interestingly, it has been found that some circRNAs are able to undergo translation both in vivo and in vitro to generate a protein [63,71]. Additionally, artificial circRNAs might direct the translation process through an internal ribosome entry site (IRES)-dependent pathway acting as mRNA, which might serve as an explanation for why circRNAs are mainly present in the cytoplasm [63]. The surprising, recently described putative function of exon–intron circRNAs (EIciRNAs) is linked to the association with polymerase II. Due to the presence of unspliced introns, such a kind of circRNA is retained within the nucleus and is shown to be enriched in the site of transcription, positively influencing the abundance of linear transcripts and the activation of the transcription [63,72]. Less understood functions of circular transcripts have also been reported, such as regulation of expression of the parental genes, serving as a scaffolds in the protein complexes, or alternative splicing control (Figure 2) [73].

The role of circRNAs has been extensively studied in various diseases, and a growing body of evidence shows neoplastic changes as one of the exceedingly numerous conditions. Circular transcripts are considered to be more often downregulated in tumor tissue compared to healthy tissue, which is explained by back-splicing machinery errors, circRNA degradation by deregulated miRNAs, or reduction of the number of circRNAs by increased cell proliferation [62,74]. Due to the ability to interact with miRNAs and RBPs, circRNAs are capable of regulating large networks of signaling pathways, leading to changes in cancer cell proliferation, invasion, and migration. Interestingly, a positive circRNA function has also been shown in in hepatocellular carcinoma (HCC), namely hsa_circ_0005986, which has been shown to inhibit cancer development by acting as an miR-129-5p sponge. Extensive research conducted in the field of circRNA profiling in various types of cancer provides evidence that circular transcripts might have a pivotal role in cancer onset and progression by global deregulation of the signaling and metabolic pathways, showing the potency to directly influence many processes, as well as indirectly via interactions with several other molecules [75].

### 3.3. SnoRNAs

Housekeeping genes (HGK), known as constitutive genes, are required for the maintenance of essential cellular functions. HGK are ubiquitously expressed in all cells of an organism under normal and pathophysiological conditions, regardless of developmental stage or exposure to external stimuli [76]. However, conserved RNAs may act as regulatory ones. One of them are small nucleolar RNAs (snoRNAs) of 60–300 nucleotides in length, predominantly concentrated in the nucleolus and Cajal bodies—the small membraneless subcompartments in the nucleus of proliferative cells [74].

There are several hundred known cellular snoRNAs [77]. In humans, some of their host genes are non-protein-coding and might act as vehicles for snoRNA synthesis. However, most snoRNAs are located in the conserved area of the introns of the housekeeping genes encoding proteins essential for ribosome biogenesis or function and transcribed by polymerase II [78]. They are chiefly located in the range of 70–90 nt upstream of the 3′ splice site. When introns are excised during splicing and form open lariats, which are subsequently degraded in exosomes, snoRNAs avoid enzymatic degradation through forming complexes with proteins, referred to as small nucleolar ribonucleoproteins (snoRNPs) [79].

SnoRNPs are essential for many cellular processes, including post-transcriptional modification of spliceosomal RNAs, chromatin maintenance, or regulation of alternative splicing [80,81,82]. They are also involved in the processing of pre-ribosomal RNA during the maturation of ribosome subunits and can be responsible for obtaining the proper tertiary structure by rRNA particles [83,84]. In addition, Cajal body-specific RNAs (scaRNAs) might control the localization of Cajal bodies in the nucleus, indicating their role in genome organization, and thus affecting gene expression (Figure 2) [85].

Two major classes of snoRNAs can be distinguished based on their characteristic sequence elements: C/D box and H/ACA box. These classes are involved in two different types of post-transcriptional modification: 2′O-ribose-methylation by the C/D box and pseudouridylation by the H/ACA box [86]. Recent reports indicate that snoRNAs can be processed into shorter molecules called sno-derived RNAs (sdRNAs) [87,88]. In animals, sdRNAs derived from the H/ACA box of snoRNAs are 20–24 nucleotides long and are located at the 3’ end, while those arising from the C/D box have a distribution of either 17–19 nucleotides in length or more than 27 nucleotides, most of which are closer to the 5′ end [89]. Functional analyses performed on different cell lines have shown that the sequences derived from both the C/D and H/ACA boxes are able to connect to the machinery responsible for RNA interference and effectively silence the genes. Positive gene regulation has been observed in some cases, which may be due to increased mRNA stability after insertion of the target sequence [88,90].

snoRNA are thought to only play a role as housekeeping molecules; the number of papers showing the involvement of snoRNAs in cancer is still growing. Recent studies have shown that changes in snoRNA expression functionally correlate with the basic cellular processes associated with carcinogenesis. For example, SNORD50 acts as a tumor suppressor in prostate and breast cancer, and SNORD113-1 is downregulated in hepatocellular carcinoma (HCC) [91,92,93]. There have also been reports of snoRNAs acting as cancer oncogenes, such as SNORA42, which is overexpressed in non-small cell lung carcinoma (NSCLC) and SNORD112-114 in a subgroup of patients with acute promyelocytic leukemia (APML) [94,95]. Differentiated expression levels of snoRNAs have also been observed in GBM, which will be examined in details below.

## 4. NcRNAs in Brain Tumor Progression

Over the past few years, great effort has been put into determining the function of ncRNAs in cancer, which has also brought to light some important discoveries in the field of brain tumors. In recent years, it has become essential to analyze patient survival, to determine the severity of tumors and treatment resistance, and, on the other hand, to analyze the molecular characteristics of tumors, taking into account altered expression pattern of various molecules, including different types of RNAs. It is worth mentioning that 40% of human lncRNAs, which amounts to 4000–20,000 transcripts, are expressed strictly in the brain [96]. Once lncRNAs were discovered to perform regulatory functions, their deregulation was the expected cause of switching from the normal to the pathological phenotype, therefore contributing to the onset of tumorigenesis [97].

There are many circRNAs that are potential cancer-driving factors as well. More than 1400 circRNAs have been identified as being differentially expressed in GBM patients, whereas 1205 have been shown to be downregulated and 206 have been shown to be upregulated [98]. A significant number of dysregulated circRNAs are involved in the ErbB and neurotrophin signaling pathways. Additionally, the extensively studied BRAF gene, which participates in the development and progression of cancers, generates circular transcript circBRAF, identified as downregulated in WHO grade III and IV patients, rather than low-grade gliomas, and is associated with poor prognosis of GBM patients [98]. There are several snoRNAs that are expressed exclusively or mainly in the CNS. Cavaille and coworkers showed that snoRNAs HBII-52 and HBI-36, which are intron-encoded in the brain-specific serotonin 2C receptor gene, are exclusively expressed in the brain, unlike all other known snoRNAs. The unexpected conclusion of the appearance of brain-specific snoRNAs and their inability to guide ribose methylations or pseudouridylation in rRNA is because of the lack of proper antisense elements, which display a peculiar function in the brain [99]. In turn, the predominant expression in the brain of snoRNAs HBII-436, HBII-438A, and HBII-438B has been found. Differentiated expression levels of snoRNAs have also been observed in GBM, which allows not only to find suppressors in this tumor, but also to differentiate gliomas according to histological grades and to reveal SNORD76 as a specific GBM biomarker [100].

**Figure 3:**
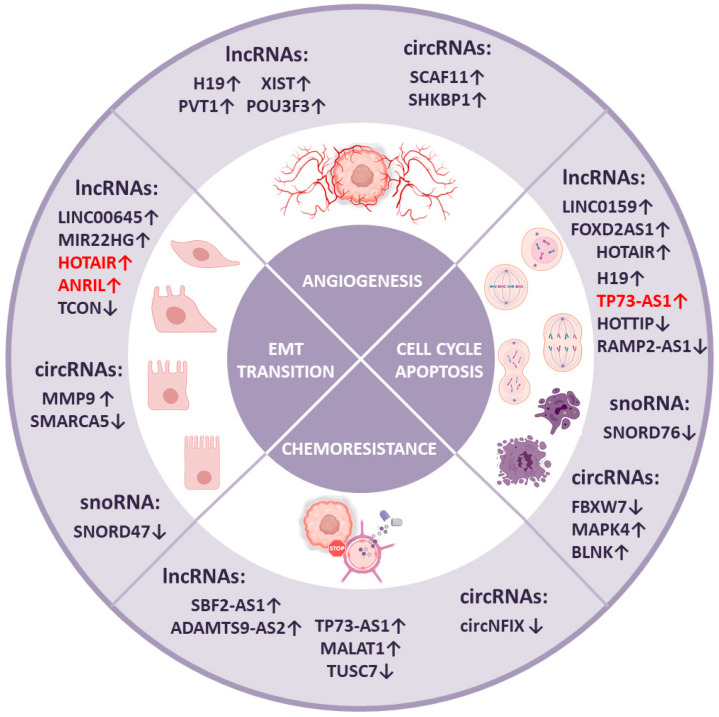
Contribution of lncRNAs, circRNAs, and snoRNAs in processes involved in brain tumors development. The arrow pointing up indicates overexpression of a particular ncRNA in brain tumor and the arrow pointing down indicates downregulation of the molecule. NcRNAs disrupted in gliomas are marked black and examples in medulloblastoma are marked red.

Every cancerous brain tumor can be characterized by several distinctive features, as extensively described in 2000 by Douglas Hannah and Robert Weinberg as the hallmarks of cancer [101]. It has also been established that many ncRNAs take part in each of these processes that contribute to cancer development. To consider the possible roles of lncRNAs, circRNAs, and snoRNAs in various aspects of brain tumor biology, we summarize existing knowledge in the context of the hallmarks of cancer presenting the ncRNAs involvement in processes like the cell cycle and apoptosis, angiogenesis, epithelial-to-mesenchymal transition, and chemoresistance, as well as their implication in cancer stem cells regulation. The summary of further described particular ncRNAs in the relation to their functions is given below (Figure 3).

### 4.1. Cell Cycle and Apoptosis

Evading apoptosis and self-sufficiency in growth signaling, along with insensitivity to the anti-growth signals of cells at the same time, is one of the crucial features of cancer. These disruptions are connected with all of the factors that regulate proper cell cycle stability. It is known that ncRNAs commonly interact with the elements involved in tumor progression [101].

One of the lncRNAs involved in cell cycle regulation is lncRNA RAMP2-AS1, which is remarkably downregulated in GBM. In the healthy brain, RAMP2-AS1 silences NOTCH3—a key factor in tumor progression associated with the induction of uncontrolled proliferation [102]. Another example of significant impact on the cell cycle is lncRNA LINC01579, which regulates both proliferation and cell apoptosis by sponging miR-139-5p, which normally targets the EIF4G2 transcript [103]. Similarly, lncRNA FOXD2-AS1 promotes glioma progression through upregulating cyclin-dependent kinase 1 by sponging miR-31 [104]. One of the best-known examples of lncRNAs is HOTAIR—an epigenetic factor that regulates the state of chromatin. HOTAIR contributes to GBM progression through proliferation by interacting with the PRC2 complex and across regulating apoptosis (via the PI3K/AKT and MEK 1/2 pathways) [105]. In the first case, HOTAIR acts as a scaffold for the PRC2 complex, which normally regulates DNA damage response. Regarding the second mechanism of HOTAIR action, it may sponge miR-326, which targets FGF1. The upregulation of FGF1 sequential leads to the stimulation of the PI3K/AKT and MEK1/2 signal pathways [106]. Another widely investigated and upregulated lncRNA is H19, which can stimulate proliferation and can inhibit apoptosis through miR-140 targeting iASPP, a well-known inhibitor of P53 [107]. Moreover, there are many other downregulated lncRNAs associated with glioma development. One of them is HOTTTIP, which inhibits BRE gene expression. Therefore, HOTTIP has been discovered to upregulate the level of the P53 protein and to repress the expression of the cyclin A and CDK2 proteins (Figure 3) [108].

circRNAs also contribute to cell cycle-dependent tumor progression. An interesting discovery is the protein FBXW7-185aa, encoded by circFBXW7, which is highly expressed in the healthy human brain. RNA-Seq of GBM samples in comparison to healthy regions of the brain, shows a reduction of the circFBXW7 in GBM patients, followed by reduced levels of the FBXW7-185aa protein. It has been shown that upregulation of FBXW7-185aa suppresses the proliferation and the cell cycle of glioma cells by reducing the half-life of c-Myc. An interesting fact is that FBXW7 linear transcripts have been shown to degrade key cellular regulators such as c-Myc, cyclin E1, c-Jun, and Notch1. These implications indicate a wide network that controls the key regulators of proliferation, which has a significant impact on gliomagenesis and tumor progression [109]. An example of upregulated circRNAs acting as an oncogenic factor is circBLNK, which sequesters miR-1236. This may possibly lead to the upregulated expression of HOXB7, although further research has to be conducted [110]. However, the circRNA circMAPK4 has been found to sponge miR-125a-3p, which, consequently, leads to the downregulation of the phosphorylation of p38/MAPK [111]. (Figure 3). On the other hand, it has been shown that SNORD76 acts as a suppressor in GBM by increasing the expression of pRb, which arrests glioma cells at the S phase of the cell cycle, which in turn may affect the expression of cell cycle-associated proteins. In addition, in orthotopic mouse models, the growth of tumors is significantly inhibited by the forced expression of SNORD76, and it is also associated with a longer survival rate of the mice (Figure 3) [100].

Likewise, a lower expression of SNORD47 has been observed in patients with GBM. In orthotopic mouse models, SNORD47 limits tumor growth and prolongs mouse survival. In lenti-SNORD47-treated groups compared to the control, minor groups of cells undergoing mitosis have been reported. It has been observed that in forced cell cycle arrest in the G2 phase (by observing the lower expression of G2 phase-specific proteins), the expression of SNORD47 significantly inhibits the proliferation of glioma cells and induces CyclinB1, CDK1, and CDC25C. Overexpression of SNORD47 leads to the reduced expression of β-catenin, which is a central component of the Wnt signaling pathway and can promote tumor cell proliferation (Figure 3) [112].

There are also ncRNAs affecting cell cycle distribution in medulloblastoma. For instance, upregulated in MB, lncRNA TP73-AS1 affects apoptosis evasion and stimulates proliferation; however, the molecular mechanism behind its action still remains unclear [113]. The individual ncRNAs involved in regulation of cell cycle and apoptosis are presented in Table 1.

### 4.2. Angiogenesis

Angiogenesis is another process essential for cancer progression, considering that nutrient and oxygen distribution are crucial for its development. Different factors have been reported to affect the growth of vascular network, including regulatory RNAs [115].

One of the lncRNAs that participates in the formation of new capillaries is H19 lncRNA, which is capable of binding to miR-29. This miRNA has been discovered to target VASH2, an important angiogenic factor. Consequently, upregulated H19 lncRNA observed in glioma tissue leads to an uncontrolled vascularization process [116]. Another widely known lncRNA, namely XIST, which was mentioned before, can enhance the angiogenesis process through increasing the level of FOXC1, CXCR7, and many others angiogenesis-related proteins by miR-137 targeting [117], followed by lncRNA PVT1, which has been found to be upregulated in gliomas and interacts with miR-186. MiR-186 inhibits glioma-conditioned human cerebral microvascular endothelial cell autophagy, thus leading to increased formation of new blood vessels [118]. Another interesting occurrence established in GBM progression connected with lncRNA level disruption is the releasing of exosomes containing lncRNA POU3F3 by glioma cells. This lncRNA is a putative factor that enhances the expression of essential angiogenesis agents such as bFGF, VEGFA, and bFGFR (Figure 3) [119].

When it comes to circRNAs, another significant axis that has been recently found is miR-421/SP1/VEGFA. This axis is one of the key players of glioma tumorigenesis and is regulated by circSCAF11, which has been shown to be significantly upregulated in GBM tissues, and its expression is positively correlated with the poor prognosis of patients [120]. Another upregulated circRNA, namely SHKBP1, has been found to increase tube formation in GBM via the miR-544a/FOXP1 and miR-379/FOXP2 pathways (Figure 3) [121]. The individual ncRNAs associated with regulation of angiogenesis are given in Table 2.

### 4.3. Epithelial-to-Mesenchymal Transition

Another hallmark of cancer is epithelial-to-mesenchymal transition (EMT), which leads to invasiveness and metastasis properties gained by the cells. It is a multistep process by which cells lose their polarity and obtain the possibility to migrate, which is a crucial step in metastasis. LncRNAs, among other ncRNAs, had been shown to play a critical role in the context of EMT in GBM [122]. By interacting with miRNA, a lncRNA named linc00645 promotes TGF-B-induced EMT by sequestering miR-205-3p, which normally downregulates ZEB1 expression. Under the circumstance of ZEB1 overabundance, (TGF)-β-induced motility of glioma cells occurs [123]. Another example of EMT-related lncRNAs is TCON, which targets the Smad/PKCα signaling pathway and simultaneously negatively regulates the progression of the malignant process, thus considered as a GBM oncosuppressor. This occurrence may be caused by lncRNA TCON complementary base pairing in Smad/PKCα mRNA, which results in blocking the translation; nevertheless, further research must be conducted [124]. Another lncRNA, MIR22HG, which undergoes high overexpression either in GBM or glioma stem-like cells and represents the host gene of miR-22-3p and miR-22-5p, promotes GBM progression by activating Wnt/β-catenin signaling via the downregulation of the *SFRP2* and *PCDH15* genes [125]. Another interesting case is matrix metalloproteinase 9 (MMP9), an enzyme involved in the degradation of the extracellular matrix under physiological conditions. However, it has been shown that the level of MMP9 is significantly increased in WHO III gliomas [126]. A study on MMP9 revealed that the MMP9 transcript has a circular form and is overexpressed in GBM tissues. Wang et al. found that the function of circMMP9 in GBM is promoting the proliferation, migration, and invasion of the cancer, acting as a sponge that targets miR-124. Interestingly, they also discovered that the circMMP9/miR-124 axis has an impact on the regulation of cyclin-dependent kinase 4 (CDK4) and aurora kinase A (AURKA), causing the upregulation and deregulation of key signaling pathways in GBM. Additionally, they established the manner of circMMP9 biogenesis, which is still unclear for a great number of circular transcripts. The biogenesis of circMMP9 depends on the eukaryotic initiation factor 4A3 (eIF4A3), which binds to the MMP3 mRNA transcript, promoting its cyclization (EIF4A3-induced circRNA MMP9 (circMMP9) acts as a sponge of miR-124 and promotes GBM cell tumorigenesis) [127]. In addition it has been discovered that circSMARCA5, which is significantly downregulated in GBM tissue, has many binding motifs for the RNA binding proteins involved in the splicing factors responsible for cell migration; thus, a shortage of circSMARCA5 leads to EMT development (Figure 3) [128].

It has been shown that artificial SNORD47 causes a significantly suppressed migration ability in U87-MG and U251 cells in wound healing assays in contrast to control cells. Furthermore, treatment with SNORD47 glioma cells leads to decreased levels of expression of N-cadherin, which is an EMT-related marker [112].

In the case of medulloblastoma, it has been established that there are many ncRNAs involved in cell mobility and metastasis. One of them—lncRNA ANRIL, which is upregulated in MB—acts as a decoy for miR-323, leading to an increase in cell viability and migration properties. Moreover, HOTAIR—a lncRNA widely described in GBM—has also been discovered to promote migration and invasion in medulloblastoma across the miR-206-YY1 axis [129,130]. The particular ncRNAs implicated in regulation of epithelial-to-mesenchymal transition process are presented in Table 3.

### 4.4. Chemoresistance

Chemoresistance is a feature that is not directly connected with the hallmarks of cancer or tumor development; nevertheless, it is the most vicious capability of cancer cells, making them insensitive to available drug treatment. NcRNAs have been recently reported to play a role in the chemoresistance of GBM. TMZ constitutes the most commonly applied first-line chemotherapeutic agent, which delays tumor progression and improves the survival rates in GBM patients. However, resistance against this compound has been observed in many cases, leading to GBM recurrence and treatment failure [131].

In several works, lncRNAs have been reported to promote this phenomenon. The new proposed mechanism in which the population of TMZ-resistant GBM cells secrete exosomes enriched in lncRNA SBF2-AS1 is the remodeling of the tumor microenvironment, thus spreading resistance to non-resistant cells. This mechanism is based on the capability of lncRNA SBF2-AS1 to sponge miR-151a-3p, which causes upregulation of its endogenous target, X-ray repair cross complementing 4 (XRCC4). This protein is a key factor in the double-strand DNA repair system in GBM cells, and its overexpression plays a crucial role in cell immortalization and in gaining chemoresistance properties [132]. Another lncRNA, named ADAMTS9-AS2, has been discovered to promote TMZ resistance by upregulating ubiquitination processes in GBM [133]. A similar effect has been observed in the case of TP73-AS1, which is overexpressed in GBM clinical samples and simultaneously contributes to the regulation of ALDH1A1 expression—a protein responsible for the TMZ resistance of GSCs [134]. Moreover, a therapeutic effect after the manipulation of the level of this lncRNA has been shown to be obtained upon MALAT1 knockdown, resulting in loss of the chemoresistance feature. This lncRNA has been discovered to sponge miRNA-101 in GBM cells and, consequently, to upregulate TMZ resistance via the MGMT and GSK3β pathways (Figure 3) [135]. On the other hand, some lncRNAs have been reported to inhibit TMZ resistance and to improve treatment outcome, such as TUSC7, which blocks TMZ resistance by interacting with miRNA-10a (Figure 3) [136].

Still, little is known about circRNA-dependent chemoresistance. The only known example is circNFIX, which is an exosomal circular RNA upregulated in the serum of TMZ-resistant patients. The mechanism behind this action is based on sponging miR-132, which leads to the development of TMZ chemoresistance, since miR-132 has been discovered to suppress glioma development. Moreover, it has been proved that exosomes can play an essential role in intracellular communication; consequently, exosomal circRNAs such as circNFIX may transit TMZ resistance from chemoresistant cancer cells to residual cells (Figure 3) [137]. The individual ncRNAs involved in regulation of cancer cell chemoresistance are given in Table 4.

### 4.5. Regulation of the Immune System

The least known but very interesting and promising aspect of cancer molecular supervision is regulation of immune compartments. Immune surveillance evasion by the tumor cells is crucial for maintenance of their proliferation potential and tumorigenesis. It has been established that the inflammatory microenvironment decreases immune response against tumor cells and induces immune escape. Immunotherapy has become a promising target for patients with cancers resistant to chemotherapy and radiation treatment or in cases for which surgical removal of the tumor is impossible [138]. There are many miRNAs identified as factors involved in immune-escape process in brain tumors. However other non-coding RNAs also appear to play important role in immune surveillance evasion; thus, further research studies must be conducted [37,139].

The GBM tumor microenvironment (TME) mainly consists of immunosuppressive cytokines and is characterized by inhibition of T-cell proliferation and effector responses, activation of FoxP3+ regulatory T-cells (Tregs), and tissue hypoxia [140]. The molecular mechanism behind this functional immunosuppression may be associated with non-coding RNAs. For instance, lncRNA CASC2c is able to interact with miR-228-3p and decrease its expression, which leads to increase secretion of coagulation factor (FX)—a factor implicated in M2 polarization of macrophages [141]. In high-grade glioma and medulloblastoma, regulation of immune compartments by ncRNA is still under-researched, but some progress was made in different types of brain cancer. In anaplastic gliomas (AG), nine-immune-related lncRNAs have been found, which has prognosis value for patients with AG [142]. Interestingly, in low-grade gliomas, survival analysis of immune-related lncRNA listed 16 lncRNAs associated with low-grade glioma prognosis, which highlights their great therapeutic potential [143].

The role of circRNA in the immune system is still the subject of intense research. While the existence of the mechanism of direct interaction of circRNA with elements of the immune system is unconfirmed, the research focused on the interaction of circRNA with miRNA involved in immunological processes. Some circular RNAs can work as molecular sponges for miRNA and, as a result, reduce the amount of miRNA available in the system. This type of interaction is well-studied for circ_002136—a circRNA able to sponge miR-138, and a potential immunomodulator in glioma. Both of these factors are also known for their role in the FUS/circ_002136/miR-138-5p/SOX13 feedback loop that moderates angiogenesis. Downregulation of miR-138 can enable glioma cells to escape immune checkpoint by restoring CTLA-4 and PD-1 expression to normal levels [144,145]. Another example of an immune response axis with circRNA as a key player is the circNFIX/miR-34a/PDL1 axis where miR-34a is a negative regulator for PD-L1—an important immune checkpoint and a promising target in cancer therapy [146]. Still, little is known about the role of snoRNAs in the immune regulatory system; however, taking into account their wide spectrum of functions, participation of these molecules in immune response evasion cannot be ruled out. 

## 5. Non-Coding RNAs in Glioma Stem Cells

Cancer stem cells (CSCs) constitute a minor immortalized subpopulation of tumor cells characterized by their self-renewal and tumorigenic capacities. There is much evidence that CSCs remain resistant to chemotherapy and radiation treatment, hence they are considered a source of metastasis in cancer progression. Due to their resistance to standard treatment, they have become a novel target in the search for molecular drugs. CSCs have also been observed in gliomas, which were appropriately named glioma stem cells (GSCs) [147]. It is known that there are many regulatory RNAs involved in the acquisitions of the stemness feature by cancer cells, the best known of which remains miRNAs. For instance, in GBM, miR-21 has been observed to target elements of the EGFR signaling pathway [148].

Since miRNAs constitute a part of the competing endogenous RNA (ceRNA) network, there are many non-coding RNAs involved in CSCs regulation. One of them is lncRNA TUG1, which affects level of miRNA-145 and negatively correlates with glioma grade [149]. Another example of a lncRNA in GSCs is CRNDE, which promotes cell proliferation and migration by sponging miR-186 and negatively regulating its level [150]. Similarly, lncRNA H19 has been found to promote cells stemness [151]. It has also been shown that lncRNA TP73-AS1 is possibly able to promote TMZ resistance in GSCs; the molecular mechanism of this action, however, still remains unclear [134]. Another mechanism affecting GSCs has been observed in the case of lncRNA FOXM1-AS, which supports nuclear interaction of the ALKBH5 protein and FOXM1 pre-mRNA, leading to enhanced FOXM1 expression and, consequently, to the regulation of GSC self-renewal and proliferation [152]. LncRNAs can also act as suppressors in GSCs—lncRNA GAS5 negatively regulates proliferation, migration, and invasion by regulating the level of miR18a-5p. It has also been proved that in gliomas, lncRNA GAS5 is significantly downregulated [153].

There is much evidence that circRNAs are also potentially implicated in the properties of CSCs. However, in the case of brain tumors, up to now, only circPTN has been found to affect the self-renewal of cells and to enhance the expression of stemness markers [154]. Nevertheless, a vast amount of evidence indicates the comprehensive role of circRNAs in proliferation, migration, and invasion, which are processes characteristic of CSCs, and consequently, the greater contribution of circRNAs cannot be denied. 

## 6. Diagnostic and Therapeutic Potential of ncRNAs

The clinical diagnosis of brain tumors relies mostly on symptoms, imaging approaches such as CT and MRI, and, finally, on histological analysis of resected tumor tissues. The advances in high-throughput technologies provide the ability of the acquisition of genome, epigenome, transcriptome, and proteome data of both bulk tumor and individual tumor cells [155]. Synergy between these two types of data leads to the generation of molecular signature that allows a better understanding the heterogeneous nature of the tumor. A good example is the transcriptome signatures that classify GBMs into four distinct subtypes among one histopathological grade, which correspond to histological and clinical sample properties. The current WHO tumor classification of the CNS already takes advantage of molecular profiling, including in the diagnosis of the IDH1/2 mutation, 1p/19q co-deletion, or histone H3K27 mutation [156]. The inclusion of these molecular signatures has a real impact on the prognosis or therapy of individual patients. As mentioned before, the main features of brain tumors are multiplicity and mutability, which lead to many difficulties with universal molecular descriptions of all cases. However, ncRNAs seem to be a promising target as potential biomarkers. 

### 6.1. Diagnostic Potential

A number of studies are based on seeking a specific pattern in meta-bases that consist of gene expression data (e.g., Gene Expression Omnibus) of gliomas and healthy brain samples. Zhang et al. carried out experiments that highlighted that several lncRNAs are capable of distinguishing between the stage and type of glioma [157]. This research, based on microarray assays, has proved that there are approximately 129 lncRNAs expressed differentially in gliomas and normal brain tissue, pointing to the stratification potential of lncRNAs.

From the whole-genome gene profiling of the CGGA cohort, the expression of lncRNA HOXA11-AS has been found to be significantly higher in high-grade glioma samples (WHO grade III/IV) than in low-grade gliomas (WHO grade I/II) [158]. The expression of HOXA11-AS in classical and mesenchymal subtypes compared to neural and proneural ones suggests that HOXA11-AS might serve as a biomarker for the identification of subtypes [158].

Similar observation was made by Jing et al. for lncRNA CRNDE in 164 gliomas and adjacent non-tumor tissues. Overexpression of CRNDE in tumor tissues was associated with a higher WHO grade, recurrence, and expansion in tumor volume, thus high expression of this lncRNA can be considered a new prognostic marker for glioma patients [159]. In turn, Zhang found that HOTAIR expression is significantly associated with tumor grade, and is also present in serum, which affords it the possibility of being a suitable diagnostic biomarker for GBM [160]. At the same time, scientists are investigating the pattern of lncRNAs that can be used as effective predictors of survival [161]. The genome-wide expression profiles based on a gene microarray performed on 80 glioma samples have shown the association of lncRNAs with overall survival of patients. This analysis has identified a prognostic signature of three lncRNAs, namely LOC441179, PON2, and USP46-AS1, which could separate GBM samples with longer overall survival from those with shorter survival [162]. The high expression of HOXA-AS3 can also be considered an independent prognostic factor related to poor prognosis and tumor grade [163]. It has also been discovered that there are lncRNAs that are highly abundant in peripheral blood samples and could be used as diagnostic biomarkers in the future. From all of the potential candidates selected from the group, 28 tumor tissue samples containing lncRNA miR210HG, which can interact with BMP1, were chosen. The expression levels of miR210HG were generally higher in sera from glioma patients than in normal tissues, and also higher in the high-risk group (WHO III or IV). It was also established that miR210HG has the sensitivity of 86.21% and the specificity of 72.41% [164]. 

Based on the lncRNAs expression profiles in gliomas, three molecular subtypes (named LncR1, LncR2, and LncR3) were also identified. These profiles were associated with gene signatures of particular glioma cells. Astroglia were rich in the LncR1 subtype, while LncR2 was enriched by the neuronal subtype and LncR3 was characterized by the oligodendrocytic gene signature [165].

There are also examples of lncRNA in MB which lead us to distinguish subgroups of this tumor more precisely. The two most heterogeneous, and otherwise closely related and difficult to distinguish, MB subgroups are groups 3 and 4. It was reported that some lncRNAs, such as ARHGEF7-AS2, lnc-HLX-1, lnc-EXPH5-2, lnc-CH25H-2, and lnc-TDRP-3, demonstrate differential expression in these two groups, and that other lncRNAs were subgroup-specific: lnc-CCL2-2 in the WNT subgroup, lnc-ABCE1-5 in the SHH subgroup, USP2-AS1 in group 3, and lnc-TBC1D16-3 in group 4 [166]. Another lncRNA, lnc-FAM84B-15 (CCAT1), was upregulated in WNT and group 3 of MB, activating the MAPK pathway and significantly stimulating proliferation and metastasis [167].

It has been determined that circRNAs exhibit an aberrant expression pattern in multiple disorders, which is becoming increasingly better identified, mainly due to RNA-Seq technology. By reason of the extensive identification of disease-related circRNAs, it is believed that they might serve as promising biomarkers and may provide a new target for the treatment. It is well known that highly complex biogenesis of circRNAs, which might be regulated at several levels by a number of molecules (both cis- and trans-factors), has an impact on cell-/tissue-specific expression patterns. Moreover, circular transcripts are abundant in saliva, exosomes, and blood samples, as well as potentially in urine or cerebrospinal fluid. All of the aforementioned reasons constitute reasonable features of putative circRNA biomarkers, which can support the traditionally used tests to increase positive diagnoses. The issue of circular transcripts as glioma biomarkers has been widely investigated, revealing a list of potential candidates for different types of gliomas, as summarized by Liu [110].

Up to now, the relevance of circRNA expression in tissues as well as the peptides or proteins encoded by circRNA for clinical parameters has been also reported. Cir-ITCH, circHIPK3, circCPA4, circ_0034642, and circ_0074362 were shown to be related to clinical severity and poor prognosis in patients with glioma [168,169,170,171,172]. The reduction of circ_0001649 was shown to be greatly associated to the larger tumor size, higher malignancy, and WHO grade. This indicates that circ_0001649 may be considered as an independent prognostic marker after surgery. Moreover, upregulation of circ_0001649 leads to apoptosis by regulating Bcl-2/caspase-3 pathway [173].

It has been also shown that circBRAF can possibly serve as an independent predictive factor with good progression-free survival and overall survival in glioma patients [98]. The promising biomarkers for predicting the prognosis of cancer patients could be also the peptides and/or proteins encoded by non-coding RNA as shown recently [174]. SHPRH-146aa, encoded by circRNA SHPRH, is abundantly reduced in GBM compared to a normal human brain. Its function is most probably linked to the reduction of tumorigenicity through protecting full-length SHPRH. The other circRNA-encoded peptide—AFBXW7-185aa—is a product of circ-FBXW7. This peptide supposes to repress glioma malignancy through USP28-induced c-Myc stabilization. Both of these molecules are related to short survival time of patients, suggesting their prognostic potential [109,175].

The diagnostic potential of circRNAs has also been preliminarily determined in MB tissues. Among 33 differentially expressed circRNAs in the MB tissues, three of them were found to be overexpressed—notably, circ-SKA3 and circ-DTL, which, by regulating host gene expression, stimulated proliferation and migration [176].

### 6.2. Therapeutic Relevance 

#### 6.2.1. ncRNAs as Therapeutic Targets

LncRNAs have been already subjected, as therapeutic targets, to clinical trials. Many epigenetic inhibitors are currently used in such research, and it has been shown that the bromodomain and extra-terminal (BET) domain proteins reduce the level of lncRNA HOTAIR [177]. Several approaches have been utilized in other types of cancer in order to target lncRNAs and to restore proper phenotypes. These methods may be used in the future as a potential method of drug delivery in GBMs. For instance, the locked nucleic acid (LNA) strategy constitutes a potent method of lncRNA activity modulation [178]. A distinction between the expression levels of lncRNAs in normal brain tissue and GBM could also indicate TMZ resistance, which would be supportive in planning a treatment approach as a tool in personalized medicine [179]. XIST/miR-29c correlation has been revealed to regulate DNA repair protein MGMT and transcription factor specificity protein 1 (SP1), participating in conferring TMZ resistance of gliomas [180].

Moreover, it has been also shown that XIST is involved in the blood–tumor barrier (BTB) permeability and glioma angiogenesis. The knockdown of XIST inhibits the expression of the transcription factor forkhead box C1 (FOXC1) and zonula occludens 2 (ZO-2) with simultaneous upregulation of miR-137. In turn, FOXC1 decreases BTB permeability by enhancing the promoter activity and expression of ZO-1 and occludin and, as a consequence, promotes glioma angiogenesis. The downregulation of XIST, then, increases the permeability of BTB, enabling the antitumor drugs delivery to a brain tumor [117]. 

In MB, it was also observed that lncRNA Nkx2-2as depress tumor suppressing targets BTG2 and LATS1 in the SHH subgroup of MB by competing with miR-103 and miR-107 and impeding cell proliferation and migration [181]. Knockdown of oncogenic lnc-IRX3-80 (CRNDE) inhibited tumor growth, significantly reduced cell proliferation, and increased the level of apoptosis in MB cell lines [182].

Of note in GBM patients is snoRNA SNORD47, which demonstrates a synergistic effect in combination with TMZ and enhances the sensitivity of gliomas to this chemotherapeutic agent. These data indicate that SNORD47 plays a key role in GBM by inhibiting the tumorigenesis process [112].

#### 6.2.2. Therapeutic Approaches Toward ncRNAs—Present State and Perspectives

One of the possible effective strategies in therapeutic targeting of tumor-specific RNAs could be an antisense oligonucleotide (ASO) technology in which short DNA oligonucleotides form a RNA–DNA hybrid with a complementary RNA target, leading to its RNAse-H-mediated degradation [183]. A notable example of such a strategy in targeting lncRNA is ASO targeting lncRNA TUG1 coupled with the drug delivery system. TUG1 is regulated by the Notch signaling pathway and is highly expressed in GSC. Downregulation of TUG1 with use of ASOs induces GSC differentiation and reduces GSC growth in vivo [184].

Circular RNAs are known to be highly stable and less prone to degradation than their linear counterparts due to the covalently closed circular structure. The unique features of circRNAs such as the structural character without 5′ caps and 3′ poly(A) tails enable their resistance to exonuclease. The half-life usually longer than 48 hours, the cell type and tissue type, the developmental-specific expression, and their high abundance in the cytoplasm of eukaryotic cells, make these molecules potentially very promising diagnostic and prognostic agents [67,185,186,187].

This property, combined with the possibility of obtaining protein-expressing circRNAs, leads to engineering novel circRNA-based therapies [188,189]. The last development in the field of circRNA expression vectors is the adeno-associated viral (AAV) vector, which can generate selected circRNAs in a tissue-specific manner with simultaneous overexpression of the protein that forms from circRNA. Results showed that overexpression of circRNA and the proteins carried by it can be targeted to specific organs such as the eyes, heart, and brain [189]. Based on these discoveries, it may be possible to obtain a circRNA-based therapeutic protein overexpression system targeting brain tumors in the near future.

In addition, the coding potential of circRNAs resulting in peptide and protein production opens a new avenue in potential GBM therapy. The peptides and proteins can also be useful in the GBM treatment responses, prognosis evaluation, and small-molecule peptide drug developments based on high specificity, activity, and lower immunogenicity. 

Moreover, nanoparticle-based approaches have been reported as strategies to possibly overcome the obstacles with the delivery of specific compounds to brain tumors across the blood–brain barrier. Therefore, targeted circRNAs and relevant peptides, as well as the lncRNAs combined with nanoparticle-based cerebral drug-delivery systems, will represent a significant new perspective for further treatment of glioma.

The other delivery system for ncRNAs that may serve not only as a target for molecular therapy but also as a tool for this type of therapy could be exosomal delivery. As would any molecular tool, ncRNAs will need a delivery system that will be reliable and stable after administration in humans. Exosomes are small extracellular vesicles that are commonly found in bodily fluids and are secreted by a wide range of human cells and tissues. Under standard conditions, exosomes can carry different types of RNA such as miRNA, circRNA, and lncRNA, but they can also be used to carry therapeutical ncRNAs to glioma cells. The first attempt in that direction was recently made with miR-302-367 encapsulated in exosomes that, after administration, can repress GBM growth [190,191]. NcRNAs as possible therapeutical targets are presented in Table 5.

## 7. Conclusions

The field of non-coding RNAs still remains underexplored and misunderstood, which makes it an interesting target for research. The wide spectrum of cellular functions may indicate their relevant role in cancer progression at the epigenetic, transcriptional, and translational levels. For this reason, ncRNAs constitute a promising target of molecular medicine; nevertheless, the expression pattern of RNAs in brain tumors remains unstable and variable, hence it is essential to search for suitable candidates that may be used as therapeutic or diagnostic tools. 

A great number of mentioned non-coding RNAs confirms their great potential; moreover, their structure may indicate that these non-coding RNAs constitute a chemically stable and long-term acting group of regulatory molecules.

Up to now, as we have summarized in this review, only a still limited number of lncRNAs and circRNAs—and even fewer snoRNAs—have been described as potential diagnostic, prognostic, and therapeutic targets in brain tumors. There is still a small number of reports showing these RNAs to be strictly related to the WHO grade, prognosis of gliomas patients, and their real potential diagnostic value. However, one can remember that a majority of the studies focus mainly on the clinical-pathological samples. The biggest challenge in the field remains waiting for the reports identifying lncRNAs, circRNAs, and snoRNAs from the bodily fluids, especially blood and CSF of brain tumor patients. The specific approaches focusing on the stability, long half-life, and abundance in, e.g., exosomes will be of great interest in the near future in order to identify the ncRNAs molecules related to the non-invasive diagnosis and stratification of the gliomas subtypes. That attempt could possibly also facilitate an early treatment assessment with high sensitivity, combined with magnetic resonance imaging or other traditional biomarkers. Therefore, the expression profiles of regulatory RNAs, determined through analyses of lncRNA, circRNAs, and snoRNAs and research of relevant roles, are urgent to further explore molecular pathology of brain tumors. In summary, the current understanding of the ncRNAs described in this review appears to be only the tip of an iceberg. The continuing efforts will make it possible to develop a novel RNA-based strategy to treat such a malignant tumor and to take advantage of their prognostic and diagnostic potential. This can possibly bring new hope for patients suffering from brain tumors.

## Figures and Tables

**Table 1:** Oncogenic or tumor-suppressive lncRNAs, circRNAs, and snoRNAs involved in the regulation of cell cycle and apoptosis.

ncRNA	Tumor	Target Name	Mechanism of Action	References
**Cell cycle and apoptosis**				
onco-ncRNAs				
lncRNA LINC01579 ↑	Glioblastoma	miR-139-5p	Upregulation of EIF4G2	[103]
lncRNA FOXD2-AS1 ↑	Glioma	miR-31	Upregulation of CDK1	[104]
lncRNA HOTAIR ↑	Glioma	miR-326	PI3K/AKT and MEK1/2 pathways activation	[106]
lncRNA HOTAIR ↑	Glioblastoma	PRC2	Cell cycle arrest evading	[105]
lncRNA H19 ↑	Glioma	miR-140	Upregulation of iASPP	[107]
circBLNK ↑	Glioma	miR-1236	Possible upregulation of HOXB7	[110]
circMAPK4 ↑	Glioma	miR-125a-3p	Downregulation of phosphorylation of p38/MAPK	[111]
lncRNA TP73-AS1 ↑	Medulloblastoma	Unknown	Unknown	[113]
antionco-ncRNAs				
lncRNA HOTTIP ↓	Glioma	BRE	Downregulation of cyclin A and CDK2; upregulation of P53	[108]
lncRNA RAMP2-AS1 ↓	Glioma	NOTCH3	Interacting with tumor promoter leading to silencing NOTCH3	[114]
circRNA FBXW7 ↓	Glioma	c-Myc	Encoding functional protein FBXW7-188aa	[109]
SNORD76 ↓	Glioblastoma	Unknown	Upregulation of pRb; downregulation of Cyclin B1, CDK1, CDC25C	[112]

**Table 2:** Oncogenic lncRNAs and circRNAs involved in the regulation of angiogenesis.

ncRNA	Tumor	Target Name	Mechanism of Action	References
**Angiogenesis**				
onco-ncRNAs				
lncRNA H19 ↑	Glioma	miR-29	Upregulation of VASH2	[116]
lncRNA XIST ↑	Glioma	miR-137	Upregulation of FOXC1 and CXCR7	[117]
lncRNA PVT1 ↑	Glioma	miR-186	Upregulation of Atg7 and Beclin1	[118]
lncRNA POU3F3 ↑	Glioma	Unknown	Upregulation of bFGF, bFGFR, and VEGFA	[119]
circRNA SCAF11 ↑	Glioma	miR-421	Upregulation of TF SP1 leading to increased expression of VEGF	[120]
circRNA SHKBP1 ↑	Glioma	miR-379 and miR-544a	Upregulation of FOXP1 and FOXP2	[121]

**Table 3:** Oncogenic or tumor-suppressive lncRNAs, circRNAs, and snoRNAs involved in the regulation of the epithelial-to-mesenchymal transition process.

ncRNA	Tumor	Target Name	Mechanism of Action	References
**Epithelial-to-mesenchymal transition**				
onco-ncRNAs				
lncRNA LINC00645 ↑	Glioma	miR-205-3p	Upregulation of ZEB1	[123]
lncRNA MIR22HG ↑	Glioblastoma	*SFRP2* and *PCDH15*	Producing miR-22-3p and miR-22-5p	[125]
lncRNA HOTAIR ↑	Medulloblastoma	miR-206	Upregulation of YY1	[129]
circRNA MMP9 ↑	Glioblastoma	miR-124	Upregulation of CDK4 and AURKA	[127]
lncRNA ANRIL ↑	Medulloblastoma	miR-323	BRI3/CDK6	[130]
antionco-ncRNAs				
lncRNA TCON ↓	Glioblastoma	Smad2 and PKCα	Downregulation of Smad2 and PKCα possibly via complementary base pairing in mRNA	[124]
circRNA SMARCA5 ↓	Glioblastoma	SRSF1	Binds RNA binding proteins involved in splicing (e.g., SRSF1)	[128]
snoRNA SNORD47 ↓	Glioblastoma	Unknown	Downregulation of N-cadherin	[112]

**Table 4:** Oncogenic or tumor-suppressive lncRNAs and circRNAs involved in regulation of cancer cell chemoresistance.

ncRNA	Tumor	Target Name	Mechanism of Action	References
**Chemoresistance**				
onco-ncRNAs				
lncRNA SBF2-AS1 ↑	Glioblastoma	miR-151a-3p	Upregulation of XRCC4	[132]
lncRNA ADAMTS9-AS2 ↑	Glioblastoma	FUS	Inhibition of MDM2-medicated FUS K48 ubiquitination	[133]
lncRNA TP73-AS1 ↑	Glioblastoma	Unknown	Upregulation of ALDH1A1	[134]
lncRNA MALAT1 ↑	Glioblastoma	miR-101	Upregulation of GSK3*β* and MGMT	[135]
circNFIX ↑	Glioma	miR-132	Modulation of ABCG2 expression	[137]
antionco-ncRNAs				
lncRNA TUSC7↓	Glioblastoma	miR-10a	Downregulation of MDR1	[136]

**Table 5:** NcRNAs as a therapy target.

ncRNA	Cancer Type	Therapeutical Strategy	References
**ncRNAs as a target for molecular therapy**			
lncRNA HOTAIR		Bromodomain and extra-terminal (BET) domain proteins	[177]
IRX3-80 (CRNDE)	MB	shRNA	[182]
lncRNA TUG1	GBM	Anti-TUG1 ASOs	[184]
lncRNA XIST		Inhibition by miRNAs	[117]
**ncRNAs as a tool for molecular therapy**			
snoRNA SNORD47	GBM	Lentiviral-based overexpression	[112]
miR-302-367	GBM	Lentiviral-based overexpression	[191]

## References

[1] Miranda-Filho A., Pineros M., Soerjomataram I., Deltour I., Bray F. (2017). Cancers of the brain and CNS: Global patterns and trends in incidence. Neuro Oncol..

[2] Brain G.B.D., Other C.N.S.C.C. (2019). Global, regional, and national burden of brain and other CNS cancer, 1990–2016: A systematic analysis for the Global Burden of Disease Study 2016. Lancet Neurol..

[3] Ferris S.P., Hofmann J.W., Solomon D.A., Perry A. (2017). Characterization of gliomas: From morphology to molecules. Virchows Arch..

[4] Stupp R., Mason W.P., van den Bent M.J., Weller M., Fisher B., Taphoorn M.J., Belanger K., Brandes A.A., Marosi C., Bogdahn U. (2005). Radiotherapy plus concomitant and adjuvant temozolomide for glioblastoma. N. Engl. J. Med..

[5] Brinkman T.M., Krasin M.J., Liu W., Armstrong G.T., Ojha R.P., Sadighi Z.S., Gupta P., Kimberg C., Srivastava D., Merchant T.E. (2016). Long-Term Neurocognitive Functioning and Social Attainment in Adult Survivors of Pediatric CNS Tumors: Results From the St Jude Lifetime Cohort Study. J. Clin. Oncol..

[6] Quail D.F., Joyce J.A. (2017). The Microenvironmental Landscape of Brain Tumors. Cancer Cell.

[7] Mackay A., Burford A., Carvalho D., Izquierdo E., Fazal-Salom J., Taylor K.R., Bjerke L., Clarke M., Vinci M., Nandhabalan M. (2017). Integrated Molecular Meta-Analysis of 1000 Pediatric High-Grade and Diffuse Intrinsic Pontine Glioma. Cancer Cell.

[8] Gilbertson R.J. (2011). Mapping cancer origins. Cell.

[9] Ling H., Vincent K., Pichler M., Fodde R., Berindan-Neagoe I., Slack F.J., Calin G.A. (2015). Junk DNA and the long non-coding RNA twist in cancer genetics. Oncogene.

[10] Wei J.W., Huang K., Yang C., Kang C.S. (2017). Non-coding RNAs as regulators in epigenetics (Review). Oncol. Rep..

[11] Mattick J.S., Makunin I.V. (2006). Non-coding RNA. Hum. Mol. Genet..

[12] Anastasiadou E., Jacob L.S., Slack F.J. (2018). Non-coding RNA networks in cancer. Nat. Rev. Cancer.

[13] De Windt L.J., Giacca M. (2018). Non-coding RNA function in stem cells and Regenerative Medicine. Noncoding RNA Res..

[14] Sakamoto N., Honma R., Sekino Y., Goto K., Sentani K., Ishikawa A., Oue N., Yasui W. (2017). Non-coding RNAs are promising targets for stem cell-based cancer therapy. Noncoding RNA Res..

[15] Turner J.D., Williamson R., Almefty K.K., Nakaji P., Porter R., Tse V., Kalani M.Y. (2010). The many roles of microRNAs in brain tumor biology. Neurosurg. Focus.

[16] Nicoloso M.S., Calin G.A. (2008). MicroRNA involvement in brain tumors: From bench to bedside. Brain Pathol..

[17] Weller M., Wick W., Aldape K., Brada M., Berger M., Pfister S.M., Nishikawa R., Rosenthal M., Wen P.Y., Stupp R. (2015). Glioma. Nat. Rev. Dis. Primers.

[18] Lakhan S.E., Harle L. (2009). Difficult diagnosis of brainstem glioblastoma multiforme in a woman: A case report and review of the literature. J. Med. Case Rep..

[19] Weller M., van den Bent M., Tonn J.C., Stupp R., Preusser M., Cohen-Jonathan-Moyal E., Henriksson R., Le Rhun E., Balana C., Chinot O. (2017). European Association for Neuro-Oncology (EANO) guideline on the diagnosis and treatment of adult astrocytic and oligodendroglial gliomas. Lancet Oncol..

[20] Chaumeil M.M., Lupo J.M., Ronen S.M. (2015). Magnetic Resonance (MR) Metabolic Imaging in Glioma. Brain Pathol..

[21] Wrensch M., Minn Y., Chew T., Bondy M., Berger M.S. (2002). Epidemiology of primary brain tumors: Current concepts and review of the literature. Neuro Oncol..

[22] Walid M.S. (2008). Prognostic factors for long-term survival after glioblastoma. Perm. J..

[23] Karcher S., Steiner H.H., Ahmadi R., Zoubaa S., Vasvari G., Bauer H., Unterberg A., Herold-Mende C. (2006). Different angiogenic phenotypes in primary and secondary glioblastomas. Int. J. Cancer.

[24] Lieberman F. (2017). Glioblastoma update: Molecular biology, diagnosis, treatment, response assessment, and translational clinical trials. F1000Research.

[25] Chamberlain M.C. (2011). Radiographic patterns of relapse in glioblastoma. J. Neurooncol..

[26] Gladson C.L., Prayson R.A., Liu W.M. (2010). The pathobiology of glioma tumors. Annu. Rev. Pathol..

[27] Gusyatiner O., Hegi M.E. (2018). Glioma epigenetics: From subclassification to novel treatment options. Semin. Cancer Biol..

[28] Yan H., Parsons D.W., Jin G., McLendon R., Rasheed B.A., Yuan W., Kos I., Batinic-Haberle I., Jones S., Riggins G.J. (2009). IDH1 and IDH2 mutations in gliomas. N. Engl. J. Med..

[29] Ohgaki H., Kleihues P. (2007). Genetic pathways to primary and secondary glioblastoma. Am. J. Pathol..

[30] Purkait S., Jha P., Sharma M.C., Suri V., Sharma M., Kale S.S., Sarkar C. (2013). CDKN2A deletion in pediatric versus adult glioblastomas and predictive value of p16 immunohistochemistry. Neuropathology.

[31] Wang Y., Leung F.C. (2004). An evaluation of new criteria for CpG islands in the human genome as gene markers. Bioinformatics.

[32] Hegi M.E., Diserens A.C., Gorlia T., Hamou M.F., de Tribolet N., Weller M., Kros J.M., Hainfellner J.A., Mason W., Mariani L. (2005). MGMT gene silencing and benefit from temozolomide in glioblastoma. N. Engl. J. Med..

[33] Verhaak R.G., Hoadley K.A., Purdom E., Wang V., Qi Y., Wilkerson M.D., Miller C.R., Ding L., Golub T., Mesirov J.P. (2010). Integrated genomic analysis identifies clinically relevant subtypes of glioblastoma characterized by abnormalities in PDGFRA, IDH1, EGFR, and NF1. Cancer Cell.

[34] Reon B.J., Anaya J., Zhang Y., Mandell J., Purow B., Abounader R., Dutta A. (2016). Expression of lncRNAs in Low-Grade Gliomas and Glioblastoma Multiforme: An In Silico Analysis. PLoS Med..

[35] Patel A.P., Tirosh I., Trombetta J.J., Shalek A.K., Gillespie S.M., Wakimoto H., Cahill D.P., Nahed B.V., Curry W.T., Martuza R.L. (2014). Single-cell RNA-seq highlights intratumoral heterogeneity in primary glioblastoma. Science.

[36] Jue T.R., McDonald K.L. (2016). The challenges associated with molecular targeted therapies for glioblastoma. J. Neurooncol..

[37] Cheng J., Meng J., Zhu L., Peng Y. (2020). Exosomal noncoding RNAs in Glioma: Biological functions and potential clinical applications. Mol. Cancer.

[38] Malbari F., Lindsay H. (2020). Genetics of Common Pediatric Brain Tumors. Pediatr. Neurol..

[39] Sturm D., Pfister S.M., Jones D.T.W. (2017). Pediatric Gliomas: Current Concepts on Diagnosis, Biology, and Clinical Management. J. Clin. Oncol..

[40] Gajjar A., Bowers D.C., Karajannis M.A., Leary S., Witt H., Gottardo N.G. (2015). Pediatric Brain Tumors: Innovative Genomic Information Is Transforming the Diagnostic and Clinical Landscape. J. Clin. Oncol..

[41] Packer R.J., Pfister S., Bouffet E., Avery R., Bandopadhayay P., Bornhorst M., Bowers D.C., Ellison D., Fangusaro J., Foreman N. (2017). Pediatric low-grade gliomas: Implications of the biologic era. Neuro Oncol..

[42] Jones D.T.W., Kieran M.W., Bouffet E., Alexandrescu S., Bandopadhayay P., Bornhorst M., Ellison D., Fangusaro J., Fisher M.J., Foreman N. (2018). Pediatric low-grade gliomas: Next biologically driven steps. Neuro Oncol..

[43] Crawford J.R., MacDonald T.J., Packer R.J. (2007). Medulloblastoma in childhood: New biological advances. Lancet Neurol..

[44] Ramaswamy V., Nor C., Taylor M.D. (2015). p53 and Meduloblastoma. Cold Spring Harb. Perspect. Med..

[45] Northcott P.A., Korshunov A., Witt H., Hielscher T., Eberhart C.G., Mack S., Bouffet E., Clifford S.C., Hawkins C.E., French P. (2011). Medulloblastoma comprises four distinct molecular variants. J. Clin. Oncol..

[46] Northcott P.A., Shih D.J., Peacock J., Garzia L., Morrissy A.S., Zichner T., Stutz A.M., Korshunov A., Reimand J., Schumacher S.E. (2012). Subgroup-specific structural variation across 1,000 medulloblastoma genomes. Nature.

[47] Taylor M.D., Northcott P.A., Korshunov A., Remke M., Cho Y.J., Clifford S.C., Eberhart C.G., Parsons D.W., Rutkowski S., Gajjar A. (2012). Molecular subgroups of medulloblastoma: The current consensus. Acta Neuropathol..

[48] Holley R.W., Apgar J., Everett G.A., Madison J.T., Marquisee M., Merrill S.H., Penswick J.R., Zamir A. (1965). STRUCTURE OF A RIBONUCLEIC ACID. Science.

[49] Eddy S.R. (2001). Non-coding RNA genes and the modern RNA world. Nat. Rev. Genet..

[50] Cao J. (2014). The functional role of long non-coding RNAs and epigenetics. Biol. Proced. Online.

[51] Esquela-Kerscher A., Slack F.J. (2006). Oncomirs-microRNAs with a role in cancer. Nat. Rev. Cancer.

[52] Kung J.T., Colognori D., Lee J.T. (2013). Long noncoding RNAs: Past, present, and future. Genetics.

[53] Beermann J., Piccoli M.T., Viereck J., Thum T. (2016). Non-coding RNAs in Development and Disease: Background, Mechanisms, and Therapeutic Approaches. Physiol. Rev..

[54] Hacisuleyman E., Goff L.A., Trapnell C., Williams A., Henao-Mejia J., Sun L., McClanahan P., Hendrickson D.G., Sauvageau M., Kelley D.R. (2014). Topological organization of multichromosomal regions by the long intergenic noncoding RNA Firre. Nat. Struct. Mol. Biol..

[55] Lin A., Li C., Xing Z., Hu Q., Liang K., Han L., Wang C., Hawke D.H., Wang S., Zhang Y. (2016). The LINK-A lncRNA activates normoxic HIF1alpha signalling in triple-negative breast cancer. Nat. Cell Biol..

[56] Mercer T.R., Dinger M.E., Mattick J.S. (2009). Long non-coding RNAs: Insights into functions. Nat. Rev. Genet..

[57] Dahariya S., Paddibhatla I., Kumar S., Raghuwanshi S., Pallepati A., Gutti R.K. (2019). Long non-coding RNA: Classification, biogenesis and functions in blood cells. Mol. Immunol..

[58] Marchese F.P., Raimondi I., Huarte M. (2017). The multidimensional mechanisms of long noncoding RNA function. Genome Biol..

[59] Cai B., Song X.Q., Cai J.P., Zhang S. (2014). HOTAIR: A cancer-related long non-coding RNA. Neoplasma.

[60] Tano K., Mizuno R., Okada T., Rakwal R., Shibato J., Masuo Y., Ijiri K., Akimitsu N. (2010). MALAT-1 enhances cell motility of lung adenocarcinoma cells by influencing the expression of motility-related genes. FEBS Lett..

[61] Leveille N., Melo C.A., Rooijers K., Diaz-Lagares A., Melo S.A., Korkmaz G., Lopes R., Moqadam F.A., Maia A.R., Wijchers P.J. (2015). Genome-wide profiling of p53-regulated enhancer RNAs uncovers a subset of enhancers controlled by a lncRNA. Nat. Commun..

[62] Greene J., Baird A.M., Brady L., Lim M., Gray S.G., McDermott R., Finn S.P. (2017). Circular RNAs: Biogenesis, Function and Role in Human Diseases. Front. Mol. Biosci..

[63] Wang Y., Wang Z. (2015). Efficient backsplicing produces translatable circular mRNAs. RNA.

[64] Barrett S.P., Wang P.L., Salzman J. (2015). Circular RNA biogenesis can proceed through an exon-containing lariat precursor. Elife.

[65] Okholm T.L.H., Nielsen M.M., Hamilton M.P., Christensen L.L., Vang S., Hedegaard J., Hansen T.B., Kjems J., Dyrskjot L., Pedersen J.S. (2017). Circular RNA expression is abundant and correlated to aggressiveness in early-stage bladder cancer. NPJ Genom. Med..

[66] Zhang X.O., Wang H.B., Zhang Y., Lu X., Chen L.L., Yang L. (2014). Complementary sequence-mediated exon circularization. Cell.

[67] Hansen T.B., Jensen T.I., Clausen B.H., Bramsen J.B., Finsen B., Damgaard C.K., Kjems J. (2013). Natural RNA circles function as efficient microRNA sponges. Nature.

[68] Lasda E., Parker R. (2014). Circular RNAs: Diversity of form and function. RNA.

[69] Patop I.L., Wust S., Kadener S. (2019). Past, present, and future of circRNAs. EMBO J..

[70] Wang Z., Lei X., Wu F.X. (2019). Identifying Cancer-Specific circRNA-RBP Binding Sites Based on Deep Learning. Molecules.

[71] Abe N., Matsumoto K., Nishihara M., Nakano Y., Shibata A., Maruyama H., Shuto S., Matsuda A., Yoshida M., Ito Y. (2015). Rolling Circle Translation of Circular RNA in Living Human Cells. Sci. Rep..

[72] Li Y., Zheng Q., Bao C., Li S., Guo W., Zhao J., Chen D., Gu J., He X., Huang S. (2015). Circular RNA is enriched and stable in exosomes: A promising biomarker for cancer diagnosis. Cell Res..

[73] Meng S., Zhou H., Feng Z., Xu Z., Tang Y., Li P., Wu M. (2017). CircRNA: Functions and properties of a novel potential biomarker for cancer. Mol. Cancer.

[74] Scotti M.M., Swanson M.S. (2016). RNA mis-splicing in disease. Nat. Rev. Genet..

[75] Fu L., Chen Q., Yao T., Li T., Ying S., Hu Y., Guo J. (2017). Hsa_circ_0005986 inhibits carcinogenesis by acting as a miR-129-5p sponge and is used as a novel biomarker for hepatocellular carcinoma. Oncotarget.

[76] Wang Z., Lyu Z., Pan L., Zeng G., Randhawa P. (2019). Defining housekeeping genes suitable for RNA-seq analysis of the human allograft kidney biopsy tissue. BMC Med. Genom..

[77] Yin Q.F., Yang L., Zhang Y., Xiang J.F., Wu Y.W., Carmichael G.G., Chen L.L. (2012). Long noncoding RNAs with snoRNA ends. Mol. Cell.

[78] Filipowicz W., Pogacic V. (2002). Biogenesis of small nucleolar ribonucleoproteins. Curr. Opin. Cell Biol..

[79] Falaleeva M., Stamm S. (2013). Processing of snoRNAs as a new source of regulatory non-coding RNAs: snoRNA fragments form a new class of functional RNAs. Bioessays.

[80] Grzechnik P., Szczepaniak S.A., Dhir S., Pastucha A., Parslow H., Matuszek Z., Mischo H.E., Kufel J., Proudfoot N.J. (2018). Nuclear fate of yeast snoRNA is determined by co-transcriptional Rnt1 cleavage. Nat. Commun..

[81] Kishore S., Khanna A., Zhang Z., Hui J., Balwierz P.J., Stefan M., Beach C., Nicholls R.D., Zavolan M., Stamm S. (2010). The snoRNA MBII-52 (SNORD 115) is processed into smaller RNAs and regulates alternative splicing. Hum. Mol. Genet..

[82] Kishore S., Stamm S. (2006). The snoRNA HBII-52 regulates alternative splicing of the serotonin receptor 2C. Science.

[83] Lafontaine D.L. (2015). Noncoding RNAs in eukaryotic ribosome biogenesis and function. Nat. Struct. Mol. Biol..

[84] McMahon M., Contreras A., Ruggero D. (2015). Small RNAs with big implications: New insights into H/ACA snoRNA function and their role in human disease. Wiley Interdiscip. Rev. RNA.

[85] Wang Q., Sawyer I.A., Sung M.H., Sturgill D., Shevtsov S.P., Pegoraro G., Hakim O., Baek S., Hager G.L., Dundr M. (2016). Cajal bodies are linked to genome conformation. Nat. Commun..

[86] Abel Y., Rederstorff M. (2019). SnoRNAs and the emerging class of sdRNAs: Multifaceted players in oncogenesis. Biochimie.

[87] Taft R.J., Glazov E.A., Lassmann T., Hayashizaki Y., Carninci P., Mattick J.S. (2009). Small RNAs derived from snoRNAs. RNA.

[88] Ender C., Krek A., Friedlander M.R., Beitzinger M., Weinmann L., Chen W., Pfeffer S., Rajewsky N., Meister G. (2008). A human snoRNA with microRNA-like functions. Mol. Cell.

[89] Scott M.S., Avolio F., Ono M., Lamond A.I., Barton G.J. (2009). Human miRNA precursors with box H/ACA snoRNA features. PLoS Comput. Biol..

[90] Brameier M., Herwig A., Reinhardt R., Walter L., Gruber J. (2011). Human box C/D snoRNAs with miRNA like functions: Expanding the range of regulatory RNAs. Nucleic Acids Res..

[91] Dong X.Y., Rodriguez C., Guo P., Sun X., Talbot J.T., Zhou W., Petros J., Li Q., Vessella R.L., Kibel A.S. (2008). SnoRNA U50 is a candidate tumor-suppressor gene at 6q14.3 with a mutation associated with clinically significant prostate cancer. Hum. Mol. Genet..

[92] Dong X.Y., Guo P., Boyd J., Sun X., Li Q., Zhou W., Dong J.T. (2009). Implication of snoRNA U50 in human breast cancer. J. Genet. Genom..

[93] Xu G., Yang F., Ding C.L., Zhao L.J., Ren H., Zhao P., Wang W., Qi Z.T. (2014). Small nucleolar RNA 113-1 suppresses tumorigenesis in hepatocellular carcinoma. Mol. Cancer.

[94] Mei Y.P., Liao J.P., Shen J., Yu L., Liu B.L., Liu L., Li R.Y., Ji L., Dorsey S.G., Jiang Z.R. (2012). Small nucleolar RNA 42 acts as an oncogene in lung tumorigenesis. Oncogene.

[95] Valleron W., Laprevotte E., Gautier E.F., Quelen C., Demur C., Delabesse E., Agirre X., Prosper F., Kiss T., Brousset P. (2012). Specific small nucleolar RNA expression profiles in acute leukemia. Leukemia.

[96] Derrien T., Johnson R., Bussotti G., Tanzer A., Djebali S., Tilgner H., Guernec G., Martin D., Merkel A., Knowles D.G. (2012). The GENCODE v7 catalog of human long noncoding RNAs: Analysis of their gene structure, evolution, and expression. Genome Res..

[97] Huarte M. (2015). The emerging role of lncRNAs in cancer. Nat. Med..

[98] Zhu J., Ye J., Zhang L., Xia L., Hu H., Jiang H., Wan Z., Sheng F., Ma Y., Li W. (2017). Differential Expression of Circular RNAs in Glioblastoma Multiforme and Its Correlation with Prognosis. Transl. Oncol..

[99] Cavaille J., Buiting K., Kiefmann M., Lalande M., Brannan C.I., Horsthemke B., Bachellerie J.P., Brosius J., Huttenhofer A. (2000). Identification of brain-specific and imprinted small nucleolar RNA genes exhibiting an unusual genomic organization. Proc. Natl. Acad. Sci. USA.

[100] Chen L., Han L., Wei J., Zhang K., Shi Z., Duan R., Li S., Zhou X., Pu P., Zhang J. (2015). SNORD76, a box C/D snoRNA, acts as a tumor suppressor in glioblastoma. Sci. Rep..

[101] Hanahan D., Weinberg R.A. (2000). The hallmarks of cancer. Cell.

[102] Liu S., Mitra R., Zhao M.M., Fan W., Eischen C.M., Yin F., Zhao Z. (2016). The Potential Roles of Long Noncoding RNAs (lncRNA) in Glioblastoma Development. Mol. Cancer Ther..

[103] Chai Y., Xie M. (2019). LINC01579 promotes cell proliferation by acting as a ceRNA of miR-139-5p to upregulate EIF4G2 expression in glioblastoma. J. Cell. Physiol..

[104] Wang J., Li B., Wang C., Luo Y., Zhao M., Chen P. (2019). Long noncoding RNA FOXD2-AS1 promotes glioma cell cycle progression and proliferation through the FOXD2-AS1/miR-31/CDK1 pathway. J. Cell. Biochem..

[105] Zhang K., Sun X., Zhou X., Han L., Chen L., Shi Z., Zhang A., Ye M., Wang Q., Liu C. (2015). Long non-coding RNA HOTAIR promotes glioblastoma cell cycle progression in an EZH2 dependent manner. Oncotarget.

[106] Ke J., Yao Y.L., Zheng J., Wang P., Liu Y.H., Ma J., Li Z., Liu X.B., Li Z.Q., Wang Z.H. (2015). Knockdown of long non-coding RNA HOTAIR inhibits malignant biological behaviors of human glioma cells via modulation of miR-326. Oncotarget.

[107] Zhao H., Peng R., Liu Q., Liu D., Du P., Yuan J., Peng G., Liao Y. (2016). The lncRNA H19 interacts with miR-140 to modulate glioma growth by targeting iASPP. Arch. Biochem. Biophys..

[108] Xu L.M., Chen L., Li F., Zhang R., Li Z.Y., Chen F.F., Jiang X.D. (2016). Over-expression of the long non-coding RNA HOTTIP inhibits glioma cell growth by BRE. J. Exp. Clin. Cancer Res..

[109] Yang Y., Gao X., Zhang M., Yan S., Sun C., Xiao F., Huang N., Yang X., Zhao K., Zhou H. (2018). Novel Role of FBXW7 Circular RNA in Repressing Glioma Tumorigenesis. J. Natl. Cancer Inst..

[110] Liu Y., Ma C., Qin X., Yu H., Shen L., Jin H. (2019). Circular RNA circ_001350 regulates glioma cell proliferation, apoptosis, and metastatic properties by acting as a miRNA sponge. J. Cell. Biochem..

[111] He J., Huang Z., He M., Liao J., Zhang Q., Wang S., Xie L., Ouyang L., Koeffler H.P., Yin D. (2020). Circular RNA MAPK4 (circ-MAPK4) inhibits cell apoptosis via MAPK signaling pathway by sponging miR-125a-3p in gliomas. Mol. Cancer.

[112] Xu B., Ye M.H., Lv S.G., Wang Q.X., Wu M.J., Xiao B., Kang C.S., Zhu X.G. (2017). SNORD47, a box C/D snoRNA, suppresses tumorigenesis in glioblastoma. Oncotarget.

[113] Varon M., Levy T., Mazor G., Ben David H., Marciano R., Krelin Y., Prasad M., Elkabets M., Pauck D., Ahmadov U. (2019). The long noncoding RNA TP73-AS1 promotes tumorigenicity of medulloblastoma cells. Int. J. Cancer.

[114] Li J., Zhu Y., Wang H., Ji X. (2018). Targeting Long Noncoding RNA in Glioma: A Pathway Perspective. Mol. Ther. Nucleic Acids.

[115] Lugano R., Ramachandran M., Dimberg A. (2020). Tumor angiogenesis: Causes, consequences, challenges and opportunities. Cell. Mol. Life Sci..

[116] Jia P., Cai H., Liu X., Chen J., Ma J., Wang P., Liu Y., Zheng J., Xue Y. (2016). Long non-coding RNA H19 regulates glioma angiogenesis and the biological behavior of glioma-associated endothelial cells by inhibiting microRNA-29a. Cancer Lett..

[117] Yu H., Xue Y., Wang P., Liu X., Ma J., Zheng J., Li Z., Li Z., Cai H., Liu Y. (2017). Knockdown of long non-coding RNA XIST increases blood-tumor barrier permeability and inhibits glioma angiogenesis by targeting miR-137. Oncogenesis.

[118] Ma Y., Wang P., Xue Y., Qu C., Zheng J., Liu X., Ma J., Liu Y. (2017). PVT1 affects growth of glioma microvascular endothelial cells by negatively regulating miR-186. Tumour Biol. J. Int. Soc. Oncodev. Biol. Med..

[119] Lang H.L., Hu G.W., Chen Y., Liu Y., Tu W., Lu Y.M., Wu L., Xu G.H. (2017). Glioma cells promote angiogenesis through the release of exosomes containing long non-coding RNA POU3F3. Eur. Rev. Med. Pharmacol. Sci..

[120] Meng Q., Li S., Liu Y., Zhang S., Jin J., Zhang Y., Guo C., Liu B., Sun Y. (2019). Circular RNA circSCAF11 Accelerates the Glioma Tumorigenesis through the miR-421/SP1/VEGFA Axis. Mol. Ther. Nucleic Acids.

[121] He Q., Zhao L., Liu Y., Liu X., Zheng J., Yu H., Cai H., Ma J., Liu L., Wang P. (2018). circ-SHKBP1 Regulates the Angiogenesis of U87 Glioma-Exposed Endothelial Cells through miR-544a/FOXP1 and miR-379/FOXP2 Pathways. Mol. Ther. Nucleic Acids.

[122] Xin S., Huang K., Zhu X.G. (2019). Non-coding RNAs: Regulators of glioma cell epithelial-mesenchymal transformation. Pathol. Res. Pract..

[123] Li C., Zheng H., Hou W., Bao H., Xiong J., Che W., Gu Y., Sun H., Liang P. (2019). Long non-coding RNA linc00645 promotes TGF-beta-induced epithelial-mesenchymal transition by regulating miR-205-3p-ZEB1 axis in glioma. Cell Death Dis..

[124] Tang C., Wang Y., Zhang L., Wang J., Wang W., Han X., Mu C., Gao D. (2020). Identification of novel LncRNA targeting Smad2/PKCalpha signal pathway to negatively regulate malignant progression of glioblastoma. J. Cell. Physiol..

[125] Han M., Wang S., Fritah S., Wang X., Zhou W., Yang N., Ni S., Huang B., Chen A., Li G. (2019). Interfering with long non-coding RNA MIR22HG processing inhibits glioblastoma progression through suppression of Wnt/beta-catenin signalling. Brain.

[126] Xue Q., Cao L., Chen X.Y., Zhao J., Gao L., Li S.Z., Fei Z. (2017). High expression of MMP9 in glioma affects cell proliferation and is associated with patient survival rates. Oncol. Lett..

[127] Wang R., Zhang S., Chen X., Li N., Li J., Jia R., Pan Y., Liang H. (2018). EIF4A3-induced circular RNA MMP9 (circMMP9) acts as a sponge of miR-124 and promotes glioblastoma multiforme cell tumorigenesis. Mol. Cancer.

[128] Barbagallo D., Caponnetto A., Cirnigliaro M., Brex D., Barbagallo C., D’Angeli F., Morrone A., Caltabiano R., Barbagallo G.M., Ragusa M. (2018). CircSMARCA5 Inhibits Migration of Glioblastoma Multiforme Cells by Regulating a Molecular Axis Involving Splicing Factors SRSF1/SRSF3/PTB. Int. J. Mol. Sci..

[129] Zhang J., Li N., Fu J., Zhou W. (2020). Long noncoding RNA HOTAIR promotes medulloblastoma growth, migration and invasion by sponging miR-1/miR-206 and targeting YY1. Biomed. Pharmacother..

[130] Zhang H., Wang X., Chen X. (2017). Potential Role of Long Non-Coding RNA ANRIL in Pediatric Medulloblastoma Through Promotion on Proliferation and Migration by Targeting miR-323. J. Cell. Biochem..

[131] Sarkaria J.N., Kitange G.J., James C.D., Plummer R., Calvert H., Weller M., Wick W. (2008). Mechanisms of chemoresistance to alkylating agents in malignant glioma. Clin. Cancer Res..

[132] Zhang Z., Yin J., Lu C., Wei Y., Zeng A., You Y. (2019). Exosomal transfer of long non-coding RNA SBF2-AS1 enhances chemoresistance to temozolomide in glioblastoma. J. Exp. Clin. Cancer Res..

[133] Yan Y., Xu Z., Chen X., Wang X., Zeng S., Zhao Z., Qian L., Li Z., Wei J., Huo L. (2019). Novel Function of lncRNA ADAMTS9-AS2 in Promoting Temozolomide Resistance in Glioblastoma via Upregulating the FUS/MDM2 Ubiquitination Axis. Front. Cell Dev. Biol..

[134] Mazor G., Levin L., Picard D., Ahmadov U., Caren H., Borkhardt A., Reifenberger G., Leprivier G., Remke M., Rotblat B. (2019). The lncRNA TP73-AS1 is linked to aggressiveness in glioblastoma and promotes temozolomide resistance in glioblastoma cancer stem cells. Cell Death Dis..

[135] Cai T., Liu Y., Xiao J. (2018). Long noncoding RNA MALAT1 knockdown reverses chemoresistance to temozolomide via promoting microRNA-101 in glioblastoma. Cancer Med..

[136] Shang C., Tang W., Pan C., Hu X., Hong Y. (2018). Long non-coding RNA TUSC7 inhibits temozolomide resistance by targeting miR-10a in glioblastoma. Cancer Chemother. Pharmacol..

[137] Ding C., Yi X., Wu X., Bu X., Wang D., Wu Z., Zhang G., Gu J., Kang D. (2020). Exosome-mediated transfer of circRNA CircNFIX enhances temozolomide resistance in glioma. Cancer Lett..

[138] Nicolini A., Ferrari P., Rossi G., Carpi A. (2018). Tumour growth and immune evasion as targets for a new strategy in advanced cancer. Endocr. Relat. Cancer.

[139] Jethwa K., Wei J., McEnery K., Heimberger A.B. (2011). miRNA-mediated immune regulation and immunotherapeutic potential in glioblastoma. Clin. Investig. (Lond.).

[140] Razavi S.M., Lee K.E., Jin B.E., Aujla P.S., Gholamin S., Li G. (2016). Immune Evasion Strategies of Glioblastoma. Front. Surg..

[141] Zhang Y., Feng J., Fu H., Liu C., Yu Z., Sun Y., She X., Li P., Zhao C., Liu Y. (2018). Coagulation Factor X Regulated by CASC2c Recruited Macrophages and Induced M2 Polarization in Glioblastoma Multiforme. Front. Immunol..

[142] Wang W., Zhao Z., Yang F., Wang H., Wu F., Liang T., Yan X., Li J., Lan Q., Wang J. (2018). An immune-related lncRNA signature for patients with anaplastic gliomas. J. Neurooncol..

[143] Li X., Meng Y. (2019). Survival analysis of immune-related lncRNA in low-grade glioma. BMC Cancer.

[144] Wei J., Nduom E.K., Kong L.Y., Hashimoto Y., Xu S., Gabrusiewicz K., Ling X., Huang N., Qiao W., Zhou S. (2016). MiR-138 exerts anti-glioma efficacy by targeting immune checkpoints. Neuro Oncol..

[145] He Z., Ruan X., Liu X., Zheng J., Liu Y., Liu L., Ma J., Shao L., Wang D., Shen S. (2019). FUS/circ_002136/miR-138-5p/SOX13 feedback loop regulates angiogenesis in Glioma. J. Exp. Clin. Cancer Res..

[146] Xu H., Zhang Y., Qi L., Ding L., Jiang H., Yu H. (2018). NFIX Circular RNA Promotes Glioma Progression by Regulating miR-34a-5p via Notch Signaling Pathway. Front. Mol. Neurosci..

[147] Najbauer J., Kraljik N., Nemeth P. (2014). Glioma stem cells: Markers, hallmarks and therapeutic targeting by metformin. Pathol. Oncol. Res..

[148] Ren Y., Zhou X., Mei M., Yuan X.B., Han L., Wang G.X., Jia Z.F., Xu P., Pu P.Y., Kang C.S. (2010). MicroRNA-21 inhibitor sensitizes human glioblastoma cells U251 (PTEN-mutant) and LN229 (PTEN-wild type) to taxol. BMC Cancer.

[149] Rani S.B., Rathod S.S., Karthik S., Kaur N., Muzumdar D., Shiras A.S. (2013). MiR-145 functions as a tumor-suppressive RNA by targeting Sox9 and adducin 3 in human glioma cells. Neuro Oncol..

[150] Zheng J., Li X.D., Wang P., Liu X.B., Xue Y.X., Hu Y., Li Z., Li Z.Q., Wang Z.H., Liu Y.H. (2015). CRNDE affects the malignant biological characteristics of human glioma stem cells by negatively regulating miR-186. Oncotarget.

[151] Jiang X., Yan Y., Hu M., Chen X., Wang Y., Dai Y., Wu D., Wang Y., Zhuang Z., Xia H. (2016). Increased level of H19 long noncoding RNA promotes invasion, angiogenesis, and stemness of glioblastoma cells. J. Neurosurg..

[152] Zhang S., Zhao B.S., Zhou A., Lin K., Zheng S., Lu Z., Chen Y., Sulman E.P., Xie K., Bogler O. (2017). m(6)A Demethylase ALKBH5 Maintains Tumorigenicity of Glioblastoma Stem-like Cells by Sustaining FOXM1 Expression and Cell Proliferation Program. Cancer Cell.

[153] Liu Q., Yu W., Zhu S., Cheng K., Xu H., Lv Y., Long X., Ma L., Huang J., Sun S. (2018). Long noncoding RNA GAS5 regulates the proliferation, migration, and invasion of glioma cells by negatively regulating miR-18a-5p. J. Cell. Physiol..

[154] Chen J., Chen T., Zhu Y., Li Y., Zhang Y., Wang Y., Li X., Xie X., Wang J., Huang M. (2019). circPTN sponges miR-145-5p/miR-330-5p to promote proliferation and stemness in glioma. J. Exp. Clin. Cancer Res..

[155] Orringer D.A., Golby A., Jolesz F. (2012). Neuronavigation in the surgical management of brain tumors: Current and future trends. Expert Rev. Med. Devices.

[156] Rodriguez F.J., Vizcaino M.A., Lin M.T. (2016). Recent Advances on the Molecular Pathology of Glial Neoplasms in Children and Adults. J. Mol. Diagn..

[157] Zhang X., Sun S., Pu J.K., Tsang A.C., Lee D., Man V.O., Lui W.M., Wong S.T., Leung G.K. (2012). Long non-coding RNA expression profiles predict clinical phenotypes in glioma. Neurobiol. Dis..

[158] Wang Q., Zhang J., Liu Y., Zhang W., Zhou J., Duan R., Pu P., Kang C., Han L. (2016). A novel cell cycle-associated lncRNA, HOXA11-AS, is transcribed from the 5-prime end of the HOXA transcript and is a biomarker of progression in glioma. Cancer Lett..

[159] Jing S.Y., Lu Y.Y., Yang J.K., Deng W.Y., Zhou Q., Jiao B.H. (2016). Expression of long non-coding RNA CRNDE in glioma and its correlation with tumor progression and patient survival. Eur. Rev. Med. Pharmacol. Sci..

[160] Zhang J.X., Han L., Bao Z.S., Wang Y.Y., Chen L.Y., Yan W., Yu S.Z., Pu P.Y., Liu N., You Y.P. (2013). HOTAIR, a cell cycle-associated long noncoding RNA and a strong predictor of survival, is preferentially expressed in classical and mesenchymal glioma. Neuro Oncol..

[161] Li M., Long S., Hu J., Wang Z., Geng C., Ou S. (2019). Systematic identification of lncRNA-based prognostic biomarkers for glioblastoma. Aging (Albany NY).

[162] Xian J., Zhang Q., Guo X., Liang X., Liu X., Feng Y. (2019). A prognostic signature based on three non-coding RNAs for prediction of the overall survival of glioma patients. FEBS Open Bio.

[163] Wu F., Zhang C., Cai J., Yang F., Liang T., Yan X., Wang H., Wang W., Chen J., Jiang T. (2017). Upregulation of long noncoding RNA HOXA-AS3 promotes tumor progression and predicts poor prognosis in glioma. Oncotarget.

[164] Min W., Dai D., Wang J., Zhang D., Zhang Y., Han G., Zhang L., Chen C., Li X., Li Y. (2016). Long Noncoding RNA miR210HG as a Potential Biomarker for the Diagnosis of Glioma. PLoS ONE.

[165] Li R., Qian J., Wang Y.Y., Zhang J.X., You Y.P. (2014). Long Noncoding RNA Profiles Reveal Three Molecular Subtypes in Glioma. CNS Neurosci. Ther..

[166] Joshi P., Jallo G., Perera R.J. (2020). In silico analysis of long non-coding RNAs in medulloblastoma and its subgroups. Neurobiol. Dis..

[167] Gao R., Zhang R., Zhang C., Zhao L., Zhang Y. (2018). Long noncoding RNA CCAT1 promotes cell proliferation and metastasis in human medulloblastoma via MAPK pathway. Tumori J..

[168] Jin P., Huang Y., Zhu P., Zou Y., Shao T., Wang O. (2018). CircRNA circHIPK3 serves as a prognostic marker to promote glioma progression by regulating miR-654/IGF2BP3 signaling. Biochem. Biophys. Res. Commun..

[169] Yang M., Li G., Fan L., Zhang G., Xu J., Zhang J. (2019). Circular RNA circ_0034642 elevates BATF3 expression and promotes cell proliferation and invasion through miR-1205 in glioma. Biochem. Biophys. Res. Commun..

[170] Peng H., Qin C., Zhang C., Su J., Xiao Q., Xiao Y., Xiao K., Liu Q. (2019). circCPA4 acts as a prognostic factor and regulates the proliferation and metastasis of glioma. J. Cell. Mol. Med..

[171] Duan X., Liu D., Wang Y., Chen Z. (2018). Circular RNA hsa_circ_0074362 Promotes Glioma Cell Proliferation, Migration, and Invasion by Attenuating the Inhibition of miR-1236-3p on HOXB7 Expression. DNA Cell Biol..

[172] Li F., Ma K., Sun M., Shi S. (2018). Identification of the tumor-suppressive function of circular RNA ITCH in glioma cells through sponging miR-214 and promoting linear ITCH expression. Am. J. Transl. Res..

[173] Wang Y., Sui X., Zhao H., Cong L., Li Y., Xin T., Guo M., Hao W. (2018). Decreased circular RNA hsa_circ_0001649 predicts unfavorable prognosis in glioma and exerts oncogenic properties in vitro and in vivo. Gene.

[174] Zhu S., Wang J., He Y., Meng N., Yan G.R. (2018). Peptides/Proteins Encoded by Non-coding RNA: A Novel Resource Bank for Drug Targets and Biomarkers. Front. Pharmacol..

[175] Zhang M., Huang N., Yang X., Luo J., Yan S., Xiao F., Chen W., Gao X., Zhao K., Zhou H. (2018). A novel protein encoded by the circular form of the SHPRH gene suppresses glioma tumorigenesis. Oncogene.

[176] Lv T., Miao Y.F., Jin K., Han S., Xu T.Q., Qiu Z.L., Zhang X.H. (2018). Dysregulated circular RNAs in medulloblastoma regulate proliferation and growth of tumor cells via host genes. Cancer Med..

[177] Pastori C., Kapranov P., Penas C., Peschansky V., Volmar C.H., Sarkaria J.N., Bregy A., Komotar R., St Laurent G., Ayad N.G. (2015). The Bromodomain protein BRD4 controls HOTAIR, a long noncoding RNA essential for glioblastoma proliferation. Proc. Natl. Acad. Sci. USA.

[178] Sarma K., Levasseur P., Aristarkhov A., Lee J.T. (2010). Locked nucleic acids (LNAs) reveal sequence requirements and kinetics of Xist RNA localization to the X chromosome. Proc. Natl. Acad. Sci. USA.

[179] Zhou K., Zhang C., Yao H., Zhang X., Zhou Y., Che Y., Huang Y. (2018). Knockdown of long non-coding RNA NEAT1 inhibits glioma cell migration and invasion via modulation of SOX2 targeted by miR-132. Mol. Cancer.

[180] Du P., Zhao H., Peng R., Liu Q., Yuan J., Peng G., Liao Y. (2017). LncRNA-XIST interacts with miR-29c to modulate the chemoresistance of glioma cell to TMZ through DNA mismatch repair pathway. Biosci. Rep..

[181] Zhang Y., Wang T., Wang S., Xiong Y., Zhang R., Zhang X., Zhao J., Yang A.G., Wang L., Jia L. (2018). Nkx2-2as Suppression Contributes to the Pathogenesis of Sonic Hedgehog Medulloblastoma. Cancer Res..

[182] Song H., Han L.M., Gao Q., Sun Y. (2016). Long non-coding RNA CRNDE promotes tumor growth in medulloblastoma. Eur. Rev. Med. Pharmacol. Sci..

[183] Zhou T., Kim Y., MacLeod A.R. (2016). Targeting Long Noncoding RNA with Antisense Oligonucleotide Technology as Cancer Therapeutics. Methods Mol. Biol..

[184] Katsushima K., Natsume A., Ohka F., Shinjo K., Hatanaka A., Ichimura N., Sato S., Takahashi S., Kimura H., Totoki Y. (2016). Targeting the Notch-regulated non-coding RNA TUG1 for glioma treatment. Nat. Commun..

[185] Jeck W.R., Sharpless N.E. (2014). Detecting and characterizing circular RNAs. Nat. Biotechnol..

[186] Barrett S.P., Salzman J. (2016). Circular RNAs: Analysis, expression and potential functions. Development.

[187] Pamudurti N.R., Bartok O., Jens M., Ashwal-Fluss R., Stottmeister C., Ruhe L., Hanan M., Wyler E., Perez-Hernandez D., Ramberger E. (2017). Translation of CircRNAs. Mol. Cell.

[188] Meganck R.M., Borchardt E.K., Castellanos Rivera R.M., Scalabrino M.L., Wilusz J.E., Marzluff W.F., Asokan A. (2018). Tissue-Dependent Expression and Translation of Circular RNAs with Recombinant AAV Vectors In Vivo. Mol. Ther. Nucleic Acids.

[189] Wesselhoeft R.A., Kowalski P.S., Anderson D.G. (2018). Engineering circular RNA for potent and stable translation in eukaryotic cells. Nat. Commun..

[190] Nair K.G.S., Ramaiyan V., Sukumaran S.K. (2018). Enhancement of drug permeability across blood brain barrier using nanoparticles in meningitis. Inflammopharmacology.

[191] Fareh M., Almairac F., Turchi L., Burel-Vandenbos F., Paquis P., Fontaine D., Lacas-Gervais S., Junier M.P., Chneiweiss H., Virolle T. (2017). Cell-based therapy using miR-302-367 expressing cells represses glioblastoma growth. Cell Death Dis..

